# Cognitive and physical activities are associated with cognitive resilience in a memory clinic cohort

**DOI:** 10.1093/braincomms/fcaf318

**Published:** 2025-09-06

**Authors:** Diana I Bocancea, Anna C van Loenhoud, Bart Kuijper, Ismael L Calandri, Colin Groot, Vikram Venkatraghavan, Casper de Boer, Mara ten Kate, Calvin Trieu, Charlotte E Teunissen, Elsmarieke van de Giessen, Anna E Leeuwis, Lisa-Marie Schlueter, Argonde C van Harten, Yolande A L Pijnenburg, Frederik Barkhof, Wiesje M van der Flier, Rik Ossenkoppele

**Affiliations:** Alzheimer Center Amsterdam, Department of Neurology, Amsterdam Neuroscience, Vrije Universiteit Amsterdam, Amsterdam UMC, Amsterdam 1081 HV, The Netherlands; Amsterdam Neuroscience, Neurodegeneration, Amsterdam 1081 HV, The Netherlands; Alzheimer Center Amsterdam, Department of Neurology, Amsterdam Neuroscience, Vrije Universiteit Amsterdam, Amsterdam UMC, Amsterdam 1081 HV, The Netherlands; Amsterdam Neuroscience, Neurodegeneration, Amsterdam 1081 HV, The Netherlands; Alzheimer Center Amsterdam, Department of Neurology, Amsterdam Neuroscience, Vrije Universiteit Amsterdam, Amsterdam UMC, Amsterdam 1081 HV, The Netherlands; Amsterdam Neuroscience, Neurodegeneration, Amsterdam 1081 HV, The Netherlands; Alzheimer Center Amsterdam, Department of Neurology, Amsterdam Neuroscience, Vrije Universiteit Amsterdam, Amsterdam UMC, Amsterdam 1081 HV, The Netherlands; Amsterdam Neuroscience, Neurodegeneration, Amsterdam 1081 HV, The Netherlands; Department of Cognitive Neurology, Fleni, Buenos Aires C1428, Argentina; Alzheimer Center Amsterdam, Department of Neurology, Amsterdam Neuroscience, Vrije Universiteit Amsterdam, Amsterdam UMC, Amsterdam 1081 HV, The Netherlands; Amsterdam Neuroscience, Neurodegeneration, Amsterdam 1081 HV, The Netherlands; Alzheimer Center Amsterdam, Department of Neurology, Amsterdam Neuroscience, Vrije Universiteit Amsterdam, Amsterdam UMC, Amsterdam 1081 HV, The Netherlands; Amsterdam Neuroscience, Neurodegeneration, Amsterdam 1081 HV, The Netherlands; Alzheimer Center Amsterdam, Department of Neurology, Amsterdam Neuroscience, Vrije Universiteit Amsterdam, Amsterdam UMC, Amsterdam 1081 HV, The Netherlands; Amsterdam Neuroscience, Neurodegeneration, Amsterdam 1081 HV, The Netherlands; Department of Radiology and Nuclear Medicine, Vrije Universiteit Amsterdam, Amsterdam UMC, Amsterdam 1081 HV, The Netherlands; Alzheimer Center Amsterdam, Department of Neurology, Amsterdam Neuroscience, Vrije Universiteit Amsterdam, Amsterdam UMC, Amsterdam 1081 HV, The Netherlands; Amsterdam Neuroscience, Neurodegeneration, Amsterdam 1081 HV, The Netherlands; Department of Laboratory Medicine, Neurochemistry Laboratory, Amsterdam Neuroscience, Program Neurodegeneration, Vrije Universiteit Amsterdam, Amsterdam University Medical Center (Amsterdam UMC), Amsterdam 1081 HV, The Netherlands; Department of Laboratory Medicine, Neurochemistry Laboratory, Amsterdam Neuroscience, Program Neurodegeneration, Vrije Universiteit Amsterdam, Amsterdam University Medical Center (Amsterdam UMC), Amsterdam 1081 HV, The Netherlands; Department of Radiology and Nuclear Medicine, Vrije Universiteit Amsterdam, Amsterdam UMC, Amsterdam 1081 HV, The Netherlands; Alzheimer Center Amsterdam, Department of Neurology, Amsterdam Neuroscience, Vrije Universiteit Amsterdam, Amsterdam UMC, Amsterdam 1081 HV, The Netherlands; Amsterdam Neuroscience, Neurodegeneration, Amsterdam 1081 HV, The Netherlands; Department of Medical Psychology, Vrije Universiteit Amsterdam, Amsterdam UMC, Amsterdam 1081 HV, The Netherlands; Alzheimer Center Amsterdam, Department of Neurology, Amsterdam Neuroscience, Vrije Universiteit Amsterdam, Amsterdam UMC, Amsterdam 1081 HV, The Netherlands; Amsterdam Neuroscience, Neurodegeneration, Amsterdam 1081 HV, The Netherlands; Alzheimer Center Amsterdam, Department of Neurology, Amsterdam Neuroscience, Vrije Universiteit Amsterdam, Amsterdam UMC, Amsterdam 1081 HV, The Netherlands; Amsterdam Neuroscience, Neurodegeneration, Amsterdam 1081 HV, The Netherlands; Alzheimer Center Amsterdam, Department of Neurology, Amsterdam Neuroscience, Vrije Universiteit Amsterdam, Amsterdam UMC, Amsterdam 1081 HV, The Netherlands; Amsterdam Neuroscience, Neurodegeneration, Amsterdam 1081 HV, The Netherlands; Department of Radiology and Nuclear Medicine, Vrije Universiteit Amsterdam, Amsterdam UMC, Amsterdam 1081 HV, The Netherlands; Queen Square Institute of Neurology and Center for Medical Image Computing, University College London, London WC1E 6BT, UK; Alzheimer Center Amsterdam, Department of Neurology, Amsterdam Neuroscience, Vrije Universiteit Amsterdam, Amsterdam UMC, Amsterdam 1081 HV, The Netherlands; Amsterdam Neuroscience, Neurodegeneration, Amsterdam 1081 HV, The Netherlands; Department of Epidemiology and Biostatistics, Vrije Universiteit Amsterdam, Amsterdam UMC, Amsterdam 1081 HV, The Netherlands; Alzheimer Center Amsterdam, Department of Neurology, Amsterdam Neuroscience, Vrije Universiteit Amsterdam, Amsterdam UMC, Amsterdam 1081 HV, The Netherlands; Amsterdam Neuroscience, Neurodegeneration, Amsterdam 1081 HV, The Netherlands; Clinical Memory Research Unit, Lund University, Lund SE-221 00, Sweden

**Keywords:** Alzheimer’s disease, cognitive resilience, lifestyle factors, cognitive activity, physical activity

## Abstract

Lifestyle factors such as participation in cognitively stimulating activities and physical activity are hypothesized to foster neural connections and enhance resilience, thereby attenuating cognitive loss in the context of Alzheimer’s disease (AD) and other neurodegenerative diseases. Nonetheless, the relationship between these factors and important clinical outcomes of cognition and brain atrophy is not well understood. We assessed cognitive and physical activity levels in a large, tertiary memory clinic cohort with various clinical and aetiological diagnoses. Furthermore, we investigated whether cognitive and physical activities relate to resilience against brain atrophy across the AD continuum. In the memory clinic-based Amsterdam Dementia Cohort (ADC), 4033 individuals completed the self-reported questionnaires to quantify their engagement in cognitive (lifetime, past and current) and physical (current) activities. Firstly, we examined differences in activity scores across diagnostic groups [i.e. Alzheimer’s and non-Alzheimer’s types of dementia, mild cognitive impairment (MCI) and subjective cognitive decline (SCD)] using linear models adjusted for age and sex. Secondly, in a subset on the AD continuum (i.e. amyloid-β-positive with SCD, MCI or Alzheimer’s dementia; *n* = 904), we used linear mixed-effects models adjusted for age and sex to assess whether cognitive and physical activities had an interactive or additive effect on concurrent cognition and rate of decline (global cognition, memory and executive functioning) at a given level of magnetic resonance imaging-based temporoparietal brain atrophy. We also tested associations with clinical progression and with mortality using Cox survival models. Lifetime and current cognitive activity, and current physical activity were generally lower in more cognitively unimpaired groups (all *P* < 0.001), while differences in past cognitive activity between diagnostic groups were not significant (*P* = 0.08). Within the AD continuum, at similar levels of temporoparietal atrophy, higher cognitive and physical activities were associated with better cognition at baseline (past cognitive activity: β_Std_ = 0.15–18, *P*_FDR_ < 0.001; and physical activity: β_Std_ = 0.9–0.11, *P*_FDR_ < 0.05). In longitudinal analyses, neither factor was related to cognitive decline nor clinical progression. Current cognitive activity [hazard ratio (HR) = 0.82 (0.73–0.92), *P*_FDR_ < 0.001] and physical activity [HR = 0.88 (0.79–0.99), *P*_FDR_ < 0.05] were associated with reduced mortality risk in the total sample, while past cognitive activity was linked to reduced mortality only in MCI [HR = 0.54 (0.36–0.8), *P*_FDR_ < 0.01]. While associations between current cognitive and physical activities with better concurrent cognitive performance might be (partially) explained by reverse causality, the observed effects of past cognitive activity suggest that early and mid-life participation in cognitively stimulating activities may provide a cognitive benefit once AD manifests.

## Teaser

Bocancea *et al*. report that higher cognitive and physical activity levels are associated with better cognition and lower mortality in individuals on the AD continuum. Although activity was not linked to slower decline, early-life cognitive engagement may contribute to resilience once symptoms emerge, supporting the importance of lifelong stimulation.

## Graphical Abstract

**Graphical Abstract:**
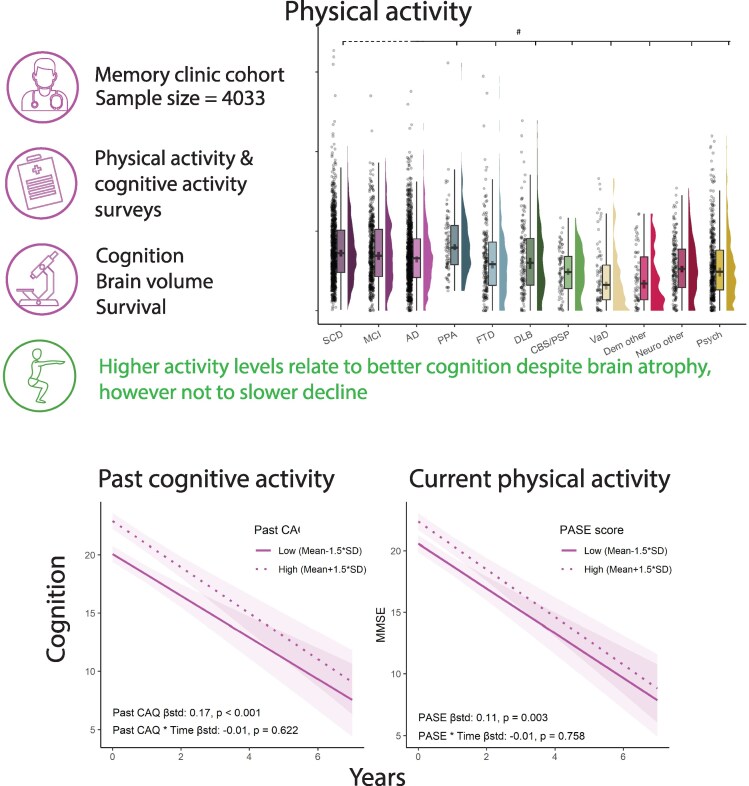

## Introduction

Cognitive resilience, grounded in the brain’s capacity for plasticity and adaptation, refers to the ability to maintain cognitive function despite neuropathological insult.^1,2^ It has emerged as a pivotal area of research in Alzheimer’s disease (AD) and other neurological disorders, as identifying modifiable factors that increase resilience could lead to non-pharmacological lifestyle interventions to preserve cognitive function during neurodegeneration.^3-5^ Life-long cognitive activities, ranging from complex problem-solving to intellectually stimulating pursuits (i.e. reading books, playing games and solving puzzles) are hypothesized to foster neural connections, enhance reserve, and consequently, sustain resilience against cognitive loss.^5,6^ Similarly, physical activity is increasingly recognized for its role in brain and cognitive health,^7^ enhancing brain perfusion,^8^ reducing inflammation,^9^ modulating neurotrophic factors and promoting synaptic plasticity and neurogenesis.^10^

Previous studies suggest that cognitive and physical activities protect against cognitive ageing and neurodegenerative disorders. For example, in a population-based elderly cohort, frequent engagement in leisure cognitive activities was associated with reduced risk of AD and mild cognitive impairment (MCI) incidence over a 5-year follow-up period,^11^ an attenuated rate of cognitive decline,^11^ and a postponed age-at-onset of AD dementia.^12^ These protective effects against dementia incidence have been replicated across multiple cohorts.^13-15^ However, the mechanistic pathways and timeline through which lifestyle can modify disease outcomes remain unclear. Some studies suggested direct neuroprotective effects on pathological markers (i.e. disease modification hypothesis), as lower levels of amyloid-β (Aβ) pathology were observed in more physically^16,17^ and cognitively active elderly,^18^ and particularly in *APOE-ɛ4* carriers.^19^ Others, however, found no relation between a cognitively active lifestyle and post-mortem AD pathology,^11,12,20^ thus the disease modification hypothesis remains unclear. Another hypothesis is that lifestyle factors mitigate the clinical manifestation of ongoing disease processes, protecting against (manifestation of) dementia through resilience, e.g. by enhancing compensatory mechanisms. A previous study showed that both cognitive and physical activities were associated with better cognition in non-demented individuals, independent of Aβ burden, hippocampal atrophy and brain glucose metabolism.^21^ While substantial research investigated these hypotheses,^3,22^ most studies primarily focused on cognitively unimpaired elderly, therefore, leaving a gap regarding more advanced clinical AD stages. Additionally, evidence for lifestyle factors’ protective effects is largely derived from community-based cohorts, with limited research on neuropathologically characterized clinical samples. Furthermore, to understand the complex interplay between (modifiable) resilience-related factors, neuropathology and clinical outcomes, longitudinal study designs are essential.

Therefore, this study has two overall goals. Firstly, we assessed the degree of cognitive and physical activities through questionnaires among individuals from a large memory clinic cohort and compared them across a range of clinical and aetiological diagnoses. We further characterized and described the relationship of these two lifestyle factors with demographics, genetic markers and imaging features. While primarily descriptive, this component of the study offers novel insights into lifetime cognitive and physical activity patterns across a variety of neurodegenerative disorders and other conditions observed in a real-world clinical setting. Secondly, to investigate the ‘resilience’ hypothesis, we examined the association of physical and cognitive activities with clinical outcomes (i.e. cognition and rate of decline, risk of progression to more advanced clinical disease stages, as well as mortality) in a subsample of individuals with confirmed AD (i.e. Aβ) pathology, adjusting for the amount of temporoparietal brain atrophy. Interactive and additive effects of cognitive and physical activities with atrophy were investigated, as modulating effects are often considered evidence of cognitive resilience.

## Materials and methods

### Participants

The sample was drawn from the ADC, a memory clinic-based cohort at the Alzheimer’s Center, Amsterdam University Medical Center.^23,24^ The sampling frame is consecutive and unselected, consisting of all patients who visited the clinic between March 2013 and August 2022 (when collection of the physical and cognitive activity questionnaires started and ended, respectively), and consented to research use of their data (*n* = 5437). As part of standardized dementia screening, patients underwent interviews on family and medical history, daily activity interferences, neuropsychological assessments, physical and neurological examination, standard laboratory tests and brain magnetic resonance imaging (MRI). They also underwent a lumbar puncture or Positron Emission Tomography (PET) imaging to determine Aβ status. Clinical diagnoses were established by consensus in multidisciplinary meetings following conventional published criteria.^25-32^

We selected all participants who (fully or partially) completed self-reported cognitive and/or physical activity questionnaires (75% responders). We collected their demographics, Aβ biomarker status, MRI data and cognitive scores closest to the questionnaire date (typically aligning with their memory clinic entry), considered the ‘baseline’ in this study. Participants were assigned the diagnosis closest to their baseline dementia screening visit or the nearest subsequent date. For most individuals, this was within a year. For 3.5%, the time difference was larger [median (IQR) = 1.7 (1.5) years]. Individuals without a clinical diagnosis (*n* = 58) were excluded.

The first part of this study (i.e. descriptive analyses) included all individuals with an available diagnosis and questionnaire data, across multiple cognitive impairment and dementia aetiologies, regardless of their biomarker status. This sample consisted of 4033 individuals, grouped in 11 diagnostic groups: subjective cognitive decline (SCD, *n* = 988), MCI (*n* = 524), AD dementia (*n* = 1208), primary progressive aphasia (PPA, *n* = 84), frontotemporal dementia (FTD, *n* = 198), dementia with Lewy bodies (DLB, *n* = 154), vascular dementia (VaD, *n* = 77), corticobasal degeneration syndrome/progressive supranuclear palsy (CBS/PSP, *n* = 78), other dementia (Dementia other, *n* = 65), other neurological conditions (Neurology other, *n* = 186) and psychiatric conditions (Pysch, *n* = 471). ‘Neurology other’ included individuals with an identifiable neurological cause of cognitive decline not related to a dementia aetiology (e.g. hydrocephalus and epilepsy). ‘Dementia other’ included individuals with dementia-level cognitive decline who did not meet the criteria for ‘Neurology other’ or for individual aetiologies. ‘Psychiatry’ included individuals with an identifiable psychiatric cause of their complaints according to Diagnosic and Statistical Manual of Mental Disorders, Fifth edition criteria (e.g. burn-out).

The second part of this study (i.e. analyses of cognitive/physical activity and cognitive resilience/clinical progression/survival) focused on an AD continuum^33^ sub-sample by including only Aβ-positive individuals, determined on CSF (drift-corrected Aβ42 < 813 pg/l^34^) or PET (positive [^18^F]flutemetamol, [^18^F]florbetaben or [^18^F]florbetapir visual read according to company guidelines or [^11^C]PiB according to published methods^35^). Participants were diagnosed with SCD [i.e. individuals presenting at our memory clinic but showing no objective cognitive deficits upon extensive neuropsychological testing, *n* = 129 (Aβ-positive)/988 (total)], MCI (*n* = 183/524) or AD dementia (*n* = 592/1208). This approach ensures that participants adhere to a biological definition of AD,^33^ while reducing heterogeneity from non-AD causes of cognitive impairment. Additionally, since resilience-related analyses required measures of brain atrophy, we excluded individuals without MRI data (38% of Aβ-positive individuals) due to unavailable scans, poor segmentation quality, or >1-year interval between MRI and questionnaire date. We further gathered all available neuropsychological follow-up data, clinical progression information and mortality status for these individuals.

### Standard protocol approvals, registrations and patient consent

Written informed consent was obtained for participation in the ADC, and study procedures were approved by the institutional review board of the Amsterdam UMC.

### Cognitive and physical activity questionnaire data

Physical activity was assessed with a Dutch version of the Physical Activity Scale for the Elderly (PASE).^36,37^ The PASE scale is a brief questionnaire suitable for the elderly and has previously shown adequate test–retest reliability^38^ and construct validity.^37,38^ The Dutch PASE version was previously validated on a Dutch sample.^39^ This 11-item questionnaire surveys activities performed over the past week in household, work-related and recreational domains. Weekly frequency and daily duration are rated on a Likert scale for activities, such as walking, sports or recreational games, etc. Total PASE scores were calculated according to the Washburn *et al*.^36^ protocol. The questionnaire item responses were first converted to measures of frequency (i.e. hours per week of activity). Overall physical activity (range: 0 to >400) was calculated as a weighted sum of the frequency scores, with higher scores indicating greater current physical activity levels.

Cognitive activity was surveyed with a modified Dutch version of the Cognitive Activity Questionnaire (CAQ).^40^ CAQ includes 25 items (rated on a five-point Likert scale) on cognitive activities (e.g. reading books, magazines, writing letters, etc.) at different life stages (ages 6, 12, 18, 40 and at current age). CAQ total scores (range: 1–5) were calculated as item averages within each time period: lifetime cognitive activity (CAQ-Lifetime; all questions), past cognitive activity (CAQ-Past; all questions excluding current) and current cognitive activity (CAQ-Current; only questions concerning current state). Higher values indicate greater cognitive activity frequency. The CAQ has shown adequate test–retest reliability.^19,40^ In our total sample, the time period-specific CAQ scores were correlated among themselves (*r*_lifetime-past CAQ_ = 0.97, *r*_past-current CAQ_ = 0.53 and *r*_lifetime-current CAQ_ = 0.70).

### Neuropsychological assessment

Neuropsychological assessment was administered with a standard test battery at baseline and follow-up visits. We used the mini-mental state examination (MMSE) to assess global cognition, and composite scores to assess memory (MEM) and executive function (EF). The domain-specific composite scores were calculated by averaging individual tests, first *z*-scored to a reference group of cognitively unimpaired Aβ-negative individuals (*n* = 440, age = 59.9 ± 6 years, 60% male, MMSE = 28.3 ± 1.5). For MEM, we included the Rey Auditory Verbal Learning Test immediate and delayed recall, and the total recall condition (A) of the Visual Association Test. For EF, we included the Digit Span Backward condition, Trail Making Test (TMT) part-B, Stroop form III and Letter Fluency tests. Time-based tests were capped at 500 s before *z*-scoring,^41^ and tests were inverted, when higher scores indicate worse performance, prior to averaging. Missing TMT-B scores were estimated using diagnosis-specific TMT B/A ratios when TMT-A data was available (AD continuum sub-sample only). Domain scores required at least two valid tests per assessment. Baseline cross-sectional cognition data corresponded to the neuropsychological assessment closest to the questionnaire date.

For the AD continuum sub-sample, we collected all cognition data available over time (i.e. for longitudinal analyses, starting 2 years prior to the questionnaire date, and all future follow-up assessments). There was variability in data availability across tests (i.e. not all individuals had all three domains measured longitudinally) and individuals had follow-up measurements at varying time intervals (see Supplementary Fig. 1).

### MRI acquisition and preprocessing

Structural T_1_-weighted images were acquired across multiple MRI scanners using standardized protocols.^42^ Volumetric scans were processed and segmented into atlas-based regions via the *Freesurfer* pipeline (http://surfer.nmr.mgh.harvard.edu/; v7.1). Segmentations were quality checked visually and scans were excluded if a substantial amount of regions were poorly segmented. Cortical thickness and surface area measures were extracted using the Desikan–Killiany atlas.^43^ MRI data were harmonized prior to analysis using the *neurocombat* algorithm,^44^ removing scanner-related variance while preserving meaningful biological variation in pre-defined variables (i.e. age, sex and diagnosis). For descriptive analyses, cortical thickness across 68 cortical regions was (surface area-weighted) averaged into a whole-brain composite region of interest (ROI). To quantify atrophy in a region representative of AD-related neurodegeneration, a temporoparietal composite ROI was calculated as the surface area-weighted average of cortical thickness in regions, including the banks of the superior temporal sulcus, inferioparietal, inferio-, middle- and superior-temporal, tansversetemporal, supramarginal, isthmuscingulate, precuneus, enthorinal and parahippocampal cortices.

### Demographics and biomarker data

Other demographic and biomarker variables-of-interest used in this study were age (at questionnaire date), sex (male/female), Aβ biomarker positivity (defined as explained above) and *APOE* genotype (ɛ4 allele positive/negative). Furthermore, education was quantified with the Dutch Verhage scale, a qualitative 7-item ordinal scale^45^ ranging from ‘1 = primary school not completed’ to ‘7 = university degree’, and converted to tertiles (based on the total sample) for further analysis, by grouping Levels 1–4 into ‘low’, Level 5 into ‘medium’ and Levels 6–7 into ‘high’ tertiles.

### Clinical progression and mortality data

Data regarding clinical progression (i.e. conversion of diagnosis, from SCD to MCI/dementia or from MCI to dementia) was available for those AD continuum individuals who visited the memory clinic more than once [median (IQR) follow-up duration = 2.32 (1.3, 3.75) years].

Furthermore, data on mortality were collected up until November 2023 through the Dutch Central Public Administration [median (IQR) follow-up duration = 4.28 (2.57, 6.33) years]. We defined follow-up duration as the interval between the questionnaire date and the date of death or, if still alive, the last date we had received information on their status (or if not available, the last date they visited the hospital).

### Missing values and imputation

Details on missing percentages across variables and diagnoses are provided in Supplementary Table 1, with missing values for biomarker data, such as Aβ status and MRI data, being most prevalent in the total sample (20% and 28%, respectively). Given the relatively large percentage, we did not impute these two variables (i.e. the presented statistics are based on the complete-case subsets).

In the total sample, some individuals were missing cross-sectional cognitive data: 2.1% were missing MMSE and 8% and 8.4% were missing the MEM or EF domain scores, respectively. For cross-sectional scores, a multiple imputation procedure was employed. Longitudinal neuropsychological domain scores were not imputed as linear mixed models (see ‘Statistical analysis’ section) were employed to model change in cognition over time, which are robust to variability in longitudinal data points.

Regarding the main variables-of-interest, i.e. activity questionnaires, 12% of participants were missing at least one item across CAQ, and 17% were missing at least one item of the PASE questionnaire. There were also missing values across demographics, *APOE* genotype and baseline cognition scores (0.4–8.4%). To minimize bias and maximize the use of item-level data, we applied a multiple imputation procedure. This approach handles missing data in CAQ/PASE, outcome and covariate variables in a single procedure, resulting in multiple imputed datasets. We followed a passive imputation scheme^46^ for the questionnaire data, using the R-based *mice* algorithm,^47^ where we imputed questionnaire missing values at the item level (as opposed to imputing the final total score) and then calculated the total scores (i.e. sum of items for CAQ; weighed sum of items for PASE). The ‘passive’ aspect of the procedure refers to the iterative nature of the method, in which the items are imputed iteratively and the total scores are passively calculated for each iteration. Furthermore, to reduce the number of predictors in the imputation model, we enforced the CAQ total score to inform the PASE item imputation, and vice versa.

Separate imputation models were fitted for different analyses. For the descriptive analyses, we imputed the questionnaire scores based on models that included as predictors age, sex, education, *APOE* genotype, diagnosis, MMSE, MEM and EF domain scores, Aβ status and the total score of the other questionnaire. Of these variables, missing values in education, *APOE* genotype and the three cognition tests (at baseline) were imputed based on the passively calculated total cognitive/physical activity scores and the remaining covariates. For the remaining analyses (i.e. the cognitive resilience and conversion/survival analyses), a similar imputation model was built. However, in this case, only the Aβ-positive AD continuum sub-sample and the temporoparietal cortical thickness ROI were included. This variable did not have missing values (since it was a selection criterion for this subsample), however, it was now included as a predictor in the imputation models for the remaining variables given that is an important predictor in the models of interest.

Given the dataset size and rate of missing values, we generated 40 imputed datasets for each imputation model (with 100 iterations to ensure correct convergence of imputed scores) and performed the statistical analyses described below within a multiple imputation framework (i.e. models are fitted to each imputed dataset and statistics are then pulled across the 40 datasets).

### Statistical analysis

All statistical analyses were done using R (v4.2.1, The R Foundation for Statistical Computing) and statistical significance was set at *P* < 0.05, two-sided.

#### Descriptive analyses

Descriptive analyses were performed in the total sample of participants to compare cognitive and physical activity levels across diagnoses and assess their association with each other as well as with demographic, genetic, cognitive and imaging variables. Group differences across diagnoses were tested with linear regression models that included each activity score (lifetime CAQ, past CAQ, current CAQ and PASE) as dependent variable and diagnosis as independent variable, while adjusting for age, sex and education. In the presence of a significant omnibus test (i.e. a multivariate Wald test, D_1_), pairwise comparisons were tested with a *post hoc* Tukey adjusted test. Activity scores were standardized to the mean and standard deviation of the total sample to calculate standardized difference estimates (we report both standardized and unstandardized coefficients). We used similar models to test for differences in CAQ and PASE scores by sex, *APOE-ɛ4* genotype and education levels (categorical variables) and test the association of activity scores with baseline age, MMSE, MEM and EF scores and whole-brain cortical thickness (continuous variables). These models were also adjusted for diagnosis (in addition to age, sex and education) as the aim was to test for overall differences and associations in the total sample. We also estimated, in similar covariate-adjusted regression models, the extent to which the different activity scores relate to each other. See Supplementary Table 4 for an overview of all models.

#### Cognitive and physical activities as determinants of cognitive resilience

To assess cognitive and physical activities as potential determinants of cognitive resilience, we operationalized resilience as the extent to which cognition remains (relatively) preserved despite the presence of brain atrophy. This definition aligns closely with the definition of cognitive reserve as described by the Collaboratory on Research Definitions for Reserve and Resilience in Cognitive Aging and Dementia (https://reserveandresilience.com/framework/). Here, we use the term ‘resilience’ because it serves as a broad descriptor that does not specify underlying mechanisms, potentially encompassing both factors that mitigate the progression of the disease (resulting in a slower rate of decline) and factors that reflect a greater premorbid reserve (leading to a higher initial cognitive level). Secondly, the term ‘resilience’ suggests a relative measure that reflects a continuum, which matches our statistical models (explained below) that infer resilience as a deviation from a normative curve of ‘expected cognitive decline for a given level of atrophy’.

To investigate the association of cognitive and physical activity levels with cognitive resilience in the AD continuum sub-sample, we modelled longitudinal cognition and assessed the effect of the questionnaire scores as a function/independent of temporoparietal atrophy. These analyses were run in the total AD continuum sample, as well as within strata by diagnosis (i.e. in the SCD, MCI and AD dementia groups separately). More specifically, we fitted linear mixed-effects models with each (longitudinal) cognitive score as outcome variable, and time (from baseline visit, in years), temporoparietal cortical thickness and the activity score of interest as independent variables. For all predictors (measured at baseline), we included interaction terms with time to assess their association with rate of decline. Random intercepts and slopes were modelled for the time variable. All individuals (i.e. those with and without follow-up available) were included in the model. We initially tested an interaction between the CAQ/PASE score and atrophy (activity*atrophy*time), to assess whether cognitive/physical activity moderated the association of atrophy with cognition/rate of decline (i.e. an interactive effect between activity and atrophy). In the absence of a significant interaction, we removed this interaction to test whether cognitive/physical activity contributes to preserving better cognition (i.e. the cross-sectional term in the model) or to slowing down rate of decline (i.e. the longitudinal term activity*time) at similar levels of atrophy (i.e. an additive effect of activity independent of atrophy). All models were controlled for age and sex (and their interaction with time) (Supplementary Table 6). Additionally, we performed sensitivity analyses in which models were also controlled for education and *APOE-ɛ4* carriership status. For certain outcomes and diagnoses, mixed-effects models with random intercepts and slopes failed to converge (due to a limited number of observations relative to random effects). In these cases, models were fitted with random intercepts only (Supplementary Table 7). For the remaining models, random slopes were included when data availability allowed and log-likelihood tests showed improved model fit. Standardized coefficients were calculated by standardizing longitudinal cognitive scores to the total sample mean and standard deviation at baseline. All other cross-sectional predictors were similarly *z*-scored with respect to the total sample. To control for multiple comparisons, false discovery rate (FDR) correction was applied across the three cognitive outcomes and all tested effects (interactions and main independent effects) within each activity questionnaire and clinical group (SCD/MCI/AD). This approach was chosen over more conservative methods that assume test independence and are likely to over penalize Type I error in the context of correlated cognitive domains and outcomes. Statistical significance was set at α < 0.05, and we report both FDR-corrected and -uncorrected results.

#### Associations with clinical progression and mortality

In the AD continuum subsample, we investigated in survival analyses whether cognitive and physical activities are predictors of disease progression and/or mortality risk, when adjusting for brain atrophy levels. We used Cox regressions among pre-dementia individuals (SCD and MCI, *n* = 312) to estimate the relationship between CAQ and PASE scores and clinical progression, defined as change in baseline diagnosis from SCD to MCI/dementia or from MCI to dementia. A model was fitted for each questionnaire score, with the activity score, temporoparietal atrophy, age and sex as predictors (and education and *APOE-ɛ4* status in sensitivity analyses). Similar Cox regressions models were fitted with mortality as outcome. These analyses were performed in the total sample and stratified by disease stage. Although we tested for interactions between the activity scores and cortical atrophy in our Cox regression models, no such effects were present. The results section will thus only describe additive effects of activity scores, based on models without interaction terms. Hazard ratios (HRs) from survival models reflect, therefore, how cognitive and physical activities affected the risk of clinical progression or mortality independent of atrophy (i.e. at similar levels of atrophy). Like previous models, we applied an FDR-correction for multiple comparisons and report both corrected and uncorrected levels of significance.

## Results

### Descriptive analyses

#### Relationships between cognitive and physical activity scores

An overview of summary statistics stratified by diagnosis is presented in Table 1 (and Supplementary Tables 2 and 3). Across the total sample, adjusting for age, sex, education and diagnosis, there was a positive association between all three cognitive activity scores (Table 2). Lifetime CAQ was most strongly associated with past CAQ (β_Std_ = 0.95, *P* < 0.001, driven by the large overlap between items), while past and current CAQ scores were correlated to a lesser extent (β_Std_ = 0.44, *P* < 0.001). Physical activity was positively associated with all three cognitive activity scores, with the largest effect size for current CAQ (β_Std_ = 0.22, *P* < 0.001).

**Table 1:** Characteristics Amsterdam Dementia Cohort—summary statistics calculated with complete case data

Variable	*N*	SCD	MCI	AD	FTD^a^	DLB	CBS/PSP	VaD	Other^b^
*N*	4033	988	524	1208	282	154	78	77	722
Age	4033	61.1 ± 8.4	65.7 ± 7.2	65.4 ± 7.6	64.4 ± 7.8	68.6 ± 5.3	66.8 ± 6.0	67.8 ± 8.2	59.5 ± 9.2
Sex, *N* females (%)	4033	408 (41)	194 (37)	637 (53)	132 (47)	33 (21)	30 (38)	28 (36)	257 (36)
Education, *N* (%)	4018								
Low		194 (20)	143 (27)	407 (34)	95 (34)	43 (28)	32 (42)	32 (42)	279 (39)
Medium		263 (27)	139 (27)	346 (29)	84 (30)	38 (25)	21 (27)	25 (32)	216 (30)
High		524 (53)	241 (46)	454 (38)	100 (36)	73 (47)	24 (31)	20 (26)	225 (31)
APOE e4, *N* positive (%)	3925	361 (38)	283 (56)	801 (68)	91 (33)	75 (50)	27 (36)	31 (40)	214 (30)
Amyloid status, *N* positive (%)	3241	174 (23)	264 (57)	1014 (95)	61 (27)	69 (62)	14 (26)	23 (38)	111 (21)
MMSE	3947	28.2 ± 1.7	26.6 ± 2.3	20.4 ± 5.3	23.6 ± 5.2	22.9 ± 4.4	24.1 ± 3.9	22.9 ± 4.6	25.7 ± 4.0
Memory	3709	−0.1 ± 0.9	−2.0 ± 1.5	−4.2 ± 2.0	−1.9 ± 1.6	−2.8 ± 1.8	−1.4 ± 1.5	−2.7 ± 1.9	−1.2 ± 1.6
Executive function	3695	−0.1 ± 0.9	−0.8 ± 0.9	−1.9 ± 1.3	−1.5 ± 1.3	−2.0 ± 1.1	−2.9 ± 1.3	−2.2 ± 1.3	−1.2 ± 1.3
Lifetime CAQ	3515	2.7 ± 0.6	2.7 ± 0.6	2.6 ± 0.6	2.6 ± 0.7	2.6 ± 0.6	2.5 ± 0.6	2.4 ± 0.7	2.4 ± 0.7
Past CAQ	3553	2.7 ± 0.7	2.6 ± 0.6	2.6 ± 0.7	2.5 ± 0.7	2.6 ± 0.6	2.4 ± 0.7	2.3 ± 0.7	2.5 ± 0.7
Current CAQ	3879	3.0 ± 0.8	3.0 ± 0.8	2.6 ± 0.8	2.6 ± 0.8	2.6 ± 0.8	2.7 ± 0.8	2.5 ± 0.9	2.4 ± 0.9
PASE score	3338	153.7 ± 81.8	144.3 ± 79.6	135.0 ± 77.3	140.6 ± 90.1	125.2 ± 91.2	96.9 ± 52.2	77.9 ± 75.4	109.0 ± 76.0
Wholebrain thickness	2885	2.4 ± 0.1	2.3 ± 0.1	2.3 ± 0.1	2.3 ± 0.1	2.3 ± 0.1	2.3 ± 0.1	2.3 ± 0.1	2.4 ± 0.1

Mean ± standard deviation, unless specified otherwise.

Summary statistics presented here were calculated based on a complete-case analyses (with varying number of available individual data points *N* per variable). Summary statistics calculated within a multiple imputation framework are shown in Supplementary Table 2.

SCD, subjective cognitive decline; MCI, mild cognitive impairment; AD, Alzheimer’s disease dementia; PPA, primary progressive aphasia; FTD, frontotemporal neurodegeneration dementia; DLB, dementia with Lewy bodies; CBS/PSP, corticobasal degeneration/progressive supranuclear palsy; VaD, vascular dementia; Other, other neurological or psychiatry conditions.

^a^The FTD group represent a combination of bvFTD, semantic variant PPA and non-fluent variant PPA. Characteristics for the different diagnostic groups are presented in Supplementary Table 3.

^b^The Other group represents a combination of dementias due to other causes (Dementia other) and other neurological and psychiatry disorders (Neuro other, Psych). Characteristics for the different diagnostic groups are presented in Supplementary Table 3.

**Table 2:** Association of cognitive and physical activities with demographic variables, cognition and cortical thickness

	CAQ Lifetime score		CAQ past score		CAQ Current score		PASE score	
	Std. estimate (SD)	*P*-value	Std. estimate (SD)	*P*-value	Std. estimate (SD)	*P*-value	Std. estimate (SD)	*P*-value
Age	0.07 (0.01)	**<0**.**001**	0.03 (0.01)	0.077	0.2 (0.02)	**<0**.**001**	−0.14 (0.02)	**<0**.**001**
Sex								
Male–female	−0.26 (0.03)	**<0**.**001**	−0.26 (0.03)	**<0**.**001**	−0.15 (0.03)	**<0**.**001**	0.21 (0.03)	**<0**.**001**
Education								
Low–medium	−0.58 (0.04)	**<0**.**001**	−0.56 (0.04)	**<0**.**001**	−0.45 (0.04)	**<0**.**001**	−0.11 (0.04)	**0**.**019**
Low–high	−1.11 (0.03)	**<0**.**001**	−1.09 (0.03)	**<0**.**001**	−0.76 (0.03)	**<0**.**001**	−0.1 (0.04)	**0**.**018**
Medium–high	−0.52 (0.03)	**<0**.**001**	−0.53 (0.03)	**<0**.**001**	−0.3 (0.04)	**<0**.**001**	0.01 (0.04)	0.981
APOE e4 status								
E4 negative–E4 positive	−0.06 (0.03)	**0**.**035**	−0.05 (0.03)	0.086	−0.07 (0.03)	**0**.**025**	−0.08 (0.03)	**0**.**016**
Cognition								
MMSE	0.15 (0.02)	**<0**.**001**	0.1 (0.02)	**<0**.**001**	0.28 (0.02)	**<0**.**001**	0.17 (0.02)	**<0**.**001**
Memory	0.04 (0.02)	**0**.**031**	0.01 (0.02)	0.656	0.13 (0.02)	**<0**.**001**	0.11 (0.02)	**<0**.**001**
Executive function	0.13 (0.02)	**<0**.**001**	0.09 (0.02)	**<0**.**001**	0.19 (0.02)	**<0**.**001**	0.08 (0.02)	**<0**.**001**
Cortical thickness								
Whole-brain	−0.01 (0.02)	0.503	−0.03 (0.02)	0.183	0.03 (0.02)	0.095	0.08 (0.02)	**0**.**001**
CAQ/PASE scores								
CAQ lifetime score	n.a.	n.a.	0.95 (0)	**<0**.**001**	0.63 (0.01)	**<0**.**001**	n.a.	n.a.
CAQ past score	n.a.	n.a.	n.a.	n.a.	0.44 (0.01)	**<0**.**001**	n.a.	n.a.
PASE score	0.2 (0.02)	**<0**.**001**	0.17 (0.02)	**<0**.**001**	0.22 (0.02)	**<0**.**001**	n.a.	n.a.

All models were adjusted for age, sex, education and diagnosis. Values in bold indicate statistically significant results (*P* < 0.05).

#### Differences in cognitive and physical activities across diagnostic groups

**Figure 1:**
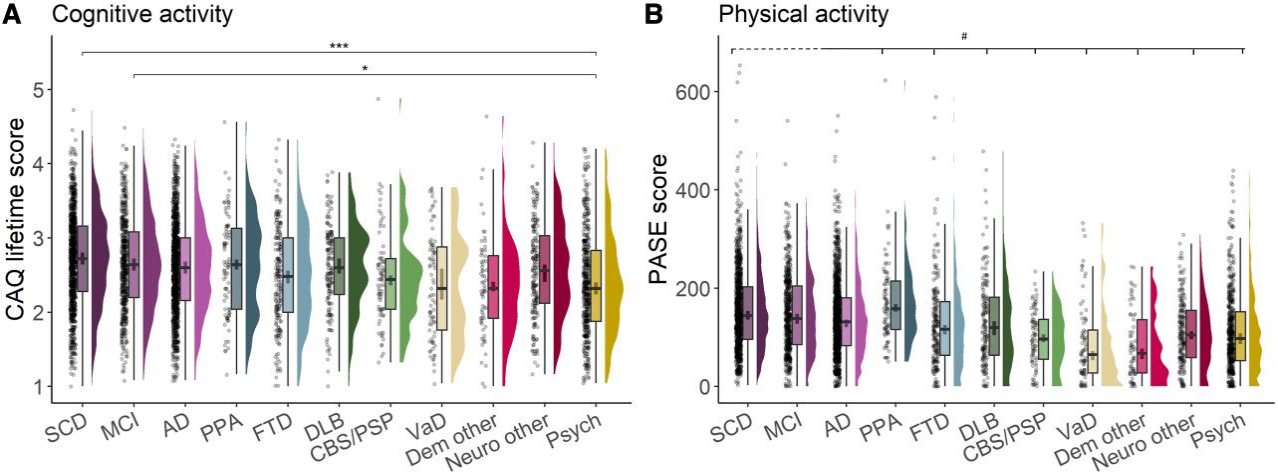
**Cognitive and physical activities across diagnostic groups in the memory clinic-based ADC cohort.** (**A**) Lifetime CAQ scores and (**B**) PASE scores. Differences across diagnostic groups were tested using linear regression models adjusted for age and sex. Sample size: *N* = 4033. Stars represent FDR-adjusted *P*-values: * = *P*_FDR_ < 0.05, ** = *P*_FDR_ < 0.01, *** = *P*_FDR_ < 0.001. # Differences in PASE scores between diagnostic groups (adjusted for age and sex): SCD > Dem other***, VaD***, CBS/PSP***, Psych***, Neuro other***, FTD*. MCI > Dem other***, VaD***, CBS/PSP***, Psych***, Neuro other***. AD > Dem other***, VaD***, CBS/PSP**, Psych***, Neuro other***. PPA > Dem other***, VaD***, CBS/PSP***, Psych***, Neuro other***, FTD**, DLB**, AD*. FTD > Dem other**, VaD*, Psych*. DLB > Dem other**, VaD**, Psych**.

Adjusting for age, sex and education, levels of lifetime CAQ (*F*(10,4004.50) = 3.73, *P* < 0.001), current CAQ (*F*(10,4003.55) = 19.62, *P* < 0.001) and PASE (*F*(10,3980.47) = 22.51, *P* < 0.001) differed across diagnostic groups, but levels of past CAQ did not (*F*(10,3980.47) = 1.67, *P* = 0.08). Adjusted pairwise group differences are shown in Supplementary Table 5. Lifetime CAQ scores were higher in SCD and MCI compared to the Psychiatry group (Fig. 1A), with no other statistically significant differences being observed between other diagnostic groups. All differences across groups in current CAQ and in (current) PASE are displayed in Fig. 1B and Supplementary Table 5. To highlight a few, SCD and MCI patients showed higher levels of both current cognitive (all *P* < 0.001) and physical activities (*P* < 0.001–0.03) compared with other dementia aetiologies, while patients with PPA showed higher levels of physical activity compared with all other dementia diagnoses (all *P* < 0.01), including AD (*P* = 0.016).

#### Association of cognitive and physical activities with demographic, genetic, cognitive and imaging variables

**Figure 2:**
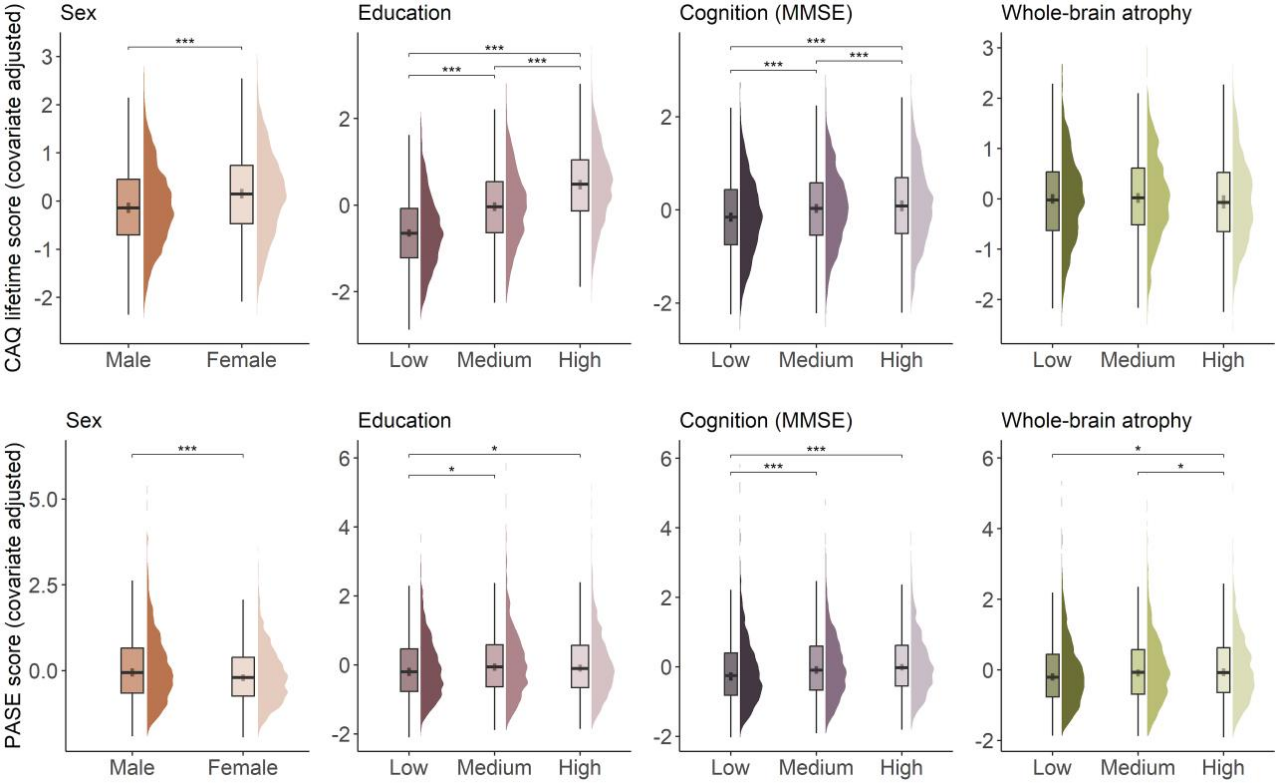
**Association of cognitive and physical activities with sex, education, cognition and whole-brain atrophy.** Lifetime CAQ and PASE scores in the total sample. Associations were estimated using linear regression models adjusted for age, sex and diagnosis. Education, cognition and whole-brain atrophy continuous variables were tertiled for plotting purposes. Sample size varies per comparison (range *N* = 3936–4033 depending on variable completeness). Stars represent *P*-values: * = *P* < 0.05, ** = *P* < 0.01, *** = *P* < 0.001.

Adjusting for other demographic variables and diagnosis, females showed higher levels of cognitive activity (across all three scores, all *P* < 0.001, with the largest difference in past CAQ β_Std_ = 0.26), whereas males showed higher levels of physical activity (β_Std_ = 0.21, *P* < 0.001) (Fig. 2). *APOE-ɛ4* carriers, compared to non-carriers, showed higher levels of current and lifetime CAQ (but not past CAQ) and PASE scores (range β_Std_ = −0.08 to 0.06, all *P* < 0.05), although these differences were smaller in magnitude (Table 2). Education was positively associated with all three CAQ scores, as well as PASE (all CAQ omnibus *F*-tests *P* < 0.001, PASE *F*-test *P* = 0.008), with increasing levels of cognitive and physical activities per increasing educational level (Table 2, Fig. 2). Older baseline age was associated with higher lifetime and current CAQ (β_Std_ = 0.2, *P* < 0.001) and with lower PASE scores (β_Std_ = −0.14, *P* < 0.001) (Table 2, Fig. 2) but was unrelated to past CAQ. Better global cognition and executive function (in models adjusted for diagnosis, i.e. within diagnostic group) were associated with higher scores across all cognitive and physical activity measures (all *P* < 0.001), while memory was positively associated with lifetime/current CAQ and PASE scores (*P* < 0.001) but not with past CAQ (Table 2, Fig. 2). When assessing associations with atrophy in the subsample with available MRI, whole-brain cortical thickness was positively associated with levels of physical activity (*P* = 0.001) but not with any of the cognitive activity scores (Table 2, Fig. 2).

### Cognitive and physical activities as determinants of cognitive resilience

**Figure 3:**
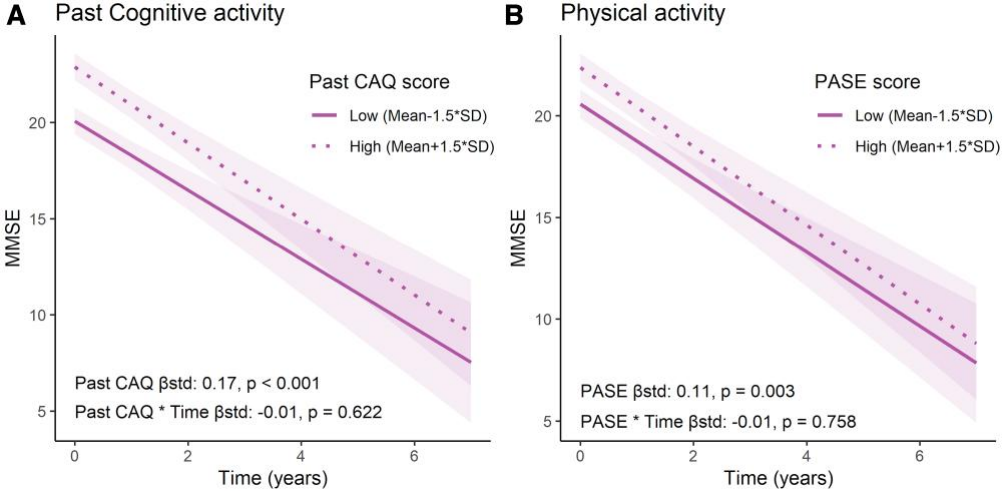
**Model-predicted trajectories of cognitive decline over time in AD dementia individuals.** (**A**) Past CAQ and (**B**) PASE effects. Predicted cognitive trajectories were derived from linear mixed-effects models adjusted for age, sex and temporoparietal atrophy, with random intercepts and slopes for time. High and low levels of activity were defined as ±1.5 SD from the mean. The data and analyses depicted in this figure pertain only to participants within the AD continuum sample. Sample: AD dementia participants within the amyloid-positive AD continuum (*N* = 592).

In models assessing their contribution to cognitive resilience (i.e. at similar levels of brain atrophy) in Aβ-positive individuals across different disease stages (Table 3), cognitive and physical activity levels showed positive additive effects on cross-sectional cognition, but no interactive effects on either cross-sectional cognition or longitudinal rate of decline. In the AD continuum, and independent of temporoparietal atrophy, higher lifetime and past CAQ scores were associated with better cognitive performance on MMSE at baseline (β_Std_ = 0.19 and β_Std_ = 0.15, both *P*_FDR_ < 0.001, Fig. 3A), primarily driven by the AD dementia group (Table 4). Notably, while a relatively small positive association was observed for memory only in the total group, a positive effect on executive function levels was observed in all three diagnostic groups (β_Std_ = 0.15–0.22, all *P*_FDR_ < 0.001, Table 4). Similarly, higher level of current CAQ was associated with better MMSE and memory in the total sample and AD subgroup, and with better executive function across all groups. Higher PASE score was also associated with better MMSE and executive function in the total sample (Fig. 3B) and the AD dementia subgroup (β_Std_ = 0.08–0.10, all *P*_FDR_ < 0.05) but not with memory (Table 4). When adjusting for education and for *APOE-ɛ4* status, a similar pattern of results remained, albeit with reduced effect sizes (Supplementary Table 8A–C).

**Table 3:** Characteristics AD continuum—summary statistics calculated with multiple imputed datasets

Participant characteristics	Total *N* = 904	SCD *N* = 129	MCI *N* = 183	AD *N* = 592
Age	65.1 ± 7.3	63.8 ± 7.5	66.1 ± 6.7	65.1 ± 7.4
Sex, *N* females (%)	462 (51)	65 (50)	84 (46)	313 (53)
Education, *N* (%)				
Low	237 (26)	21 (16)	36 (20)	180 (30)
Medium	256 (28)	33 (26)	41 (22)	182 (31)
High	411 (45)	75 (58)	106 (58)	230 (39)
APOE e4, *N* positive (%)	641 (71)	86 (67)	138 (75)	417 (70)
MMSE	23.0 ± 5.4	28.2 ± 1.7	26.4 ± 2.3	20.8 ± 5.3
Memory	−3.1 ± 2.3	−0.2 ± 0.8	−2.2 ± 1.4	−4.1 ± 2.0
Executive function	−1.3 ± 1.4	−0.1 ± 0.8	−0.5 ± 0.8	−1.9 ± 1.4
Lifetime CAQ	2.7 ± 0.6	2.8 ± 0.6	2.8 ± 0.6	2.6 ± 0.6
Past CAQ	2.6 ± 0.7	2.7 ± 0.6	2.7 ± 0.6	2.6 ± 0.7
Current CAQ	2.8 ± 0.8	3.1 ± 0.7	3.1 ± 0.7	2.7 ± 0.8
PASE score	148.4 ± 77.2	154.0 ± 78.5	162.3 ± 79.9	142.9 ± 75.5
Wholebrain thickness	2.3 ± 0.1	2.4 ± 0.1	2.3 ± 0.1	2.3 ± 0.1
Temporoparietal thickness	2.4 ± 0.1	2.5 ± 0.1	2.4 ± 0.1	2.3 ± 0.1
Longitudinal cognition, median (IQR)				
MMSE follow up (years)	1.1 (0.04, 2.7)	2.4 (0.2, 4.5)	2.1 (0.3, 3.4)	0.9 (0.01, 2.0)
Memory follow-up (years)	1.0 (0.03, 2.4)	2.2 (0.2, 4.1)	2.0 (0.1, 3.3)	0.2 (0.00, 1.9)
Executive function follow-up (years)	0.2 (0.00, 2.1)	2.3 (0.2, 4.2)	1.9 (0.1, 3.1)	0.1 (0.00, 1.1)
Clinical progression				
Diagnostic conversions, *N* (%)	116 (37)	37 (29)	79 (43)	-
Follow-up (years), median (IQR)	1.5 (0.00, 3.2)	2.0 (0.00, 4.0)	1.2 (0.00, 2.8)	-
Mortality				
Deceased during follow-up, *N* (%)	301 (33)	12 (9.3)	39 (21)	250 (42)
Follow-up (years), median (IQR)	4.3 (2.6, 6.3)	4.1 (2.5, 6.3)	4.8 (2.5, 6.5)	4.6 (3.2, 6.4)

The data depicted in this table pertain only to participants within the amyloid-positive AD continuum sample.

**Table 4:** Main results in the AD continuum sample

			MMSE^a^		Memory^a^		Executive function^a^		Mortality^b^		Clinical progression to MCI/dementia^b^	
			Estimate [CI]	*P*	Estimate [CI]	*P*	Estimate [CI]	*P*	HR [CI]	*P*	HR [CI]	*P*
β Questionnaire score	Lifetime CAQ	All	0.195 [0.142–0.247]	**<0**.001***	0.102 [0.039–0.164]	**0**.001**	0.222 [0.163–0.281]	**<0**.001***	0.88 [0.78–0.98]	**0**.02*	1.03 [0.83–1.27]	0.81
		SCD	0.035 [−0.014–0.083]	0.165	0.057 [−0.01–0.124]	0.097	0.194 [0.107–0.282]	**<0**.001***	0.98 [0.45–2.13]	0.96	0.96 [0.63–1.46]	0.85
		MCI	0.026 [−0.038–0.09]	0.427	−0.05 [−0.151–0.052]	0.338	0.161 [0.09–0.232]	**<0**.001***	0.53 [0.35–0.8]	**<0**.001**	1.04 [0.81–1.33]	0.76
		AD	0.217 [0.147–0.286]	**<0**.001***	0.053 [−0.017–0.123]	0.137	0.201 [0.123–0.28]	**<0**.001***	0.97 [0.86–1.1]	0.64	-	-
	Past CAQ	All	0.152 [0.099–0.205]	**<0**.001***	0.077 [0.015–0.139]	**0**.**016**	0.182 [0.123–0.242]	**<0**.001***	0.91 [0.81–1.02]	0.09	1.07 [0.86–1.31]	0.55
		SCD	0.03 [−0.018–0.078]	0.218	0.054 [−0.012–0.12]	0.109	0.177 [0.09–0.264]	**<0**.001***	1.02 [0.48–2.17]	0.95	0.99 [0.66–1.48]	0.95
		MCI	0.02 [−0.042–0.083]	0.523	−0.054 [−0.153–0.046]	0.288	0.15 [0.08–0.219]	**<0**.001***	0.54 [0.36–0.8]	**<0**.001**	1.07 [0.84–1.37]	0.56
		AD	0.171 [0.102–0.242]	**<0**.001***	0.041 [−0.03–0.11]	0.260	0.164 [0.084–0.243]	**<0**.001***	1 [0.88–1.14]	0.99	-	-
	Current CAQ	All	0.265 [0.213–0.318]	**<0**.001***	0.148 [0.084–0.212]	**<0**.001***	0.277 [0.217–0.337]	**<0**.001***	0.82 [0.73–0.92]	**<0**.001***	0.86 [0.68–1.09]	0.20
		SCD	0.045 [−0.013–0.102]	0.127	0.053 [−0.027–0.134]	0.192	0.216 [0.111–0.32]	**<0**.001***	0.8 [0.3–2.11]	0.60	0.85 [0.52–1.41]	0.53
		MCI	0.029 [−0.04–0.098]	0.408	−0.02 [−0.131–0.092]	0.732	0.139 [0.061–0.216]	**<0**.001**	0.77 [0.51–1.16]	0.20	0.87 [0.66–1.15]	0.33
		AD	0.279 [0.211–0.347]	**<0**.001***	0.075 [0.005–0.146]	**0**.**037**	0.252 [0.173–0.33]	**<0**.001***	0.88 [0.77–1]	0.06	-	-
	PASE	All	0.079 [0.027–0.131]	**0**.003*	0.025 [−0.035–0.086]	0.417	0.083 [0.025–0.141]	**0**.005*	0.88 [0.79–0.99]	**0**.04*	0.94 [0.77–1.14]	0.52
		SCD	−0.016 [−0.059–0.028]	0.479	0.019 [−0.041–0.08]	0.53	0.039 [−0.044–0.122]	0.356	0.94 [0.36–2.48]	0.88	0.93 [0.6–1.42]	0.72
		MCI	−0.02 [−0.077–0.038]	0.502	−0.05 [−0.141–0.041]	0.276	−0.011 [−0.078–0.057]	0.76	1.02 [0.75–1.39]	0.91	0.92 [0.73–1.16]	0.47
		AD	0.108 [0.036–0.179]	**0**.003*	0.024 [−0.046–0.094]	0.499	0.107 [0.028–0.185]	**0**.008*	0.87 [0.76–0.99]	**0**.03*	-	-

Values in bold indicate statistically significant results (*P* < 0.05).

Stars represent FDR adjusted *P*-values: * = *P*_FDR_ < 0.05, ** = *P*_FDR_ < 0.01, *** = *P*_FDR_ < 0.001.

Analyses presented in this table were conducted exclusively within the amyloid-positive AD continuum sample.

^a^Cross-sectional main effects on cognition independent of temporoparietal cortical thickness. Coefficients from linear mixed-effect models, adjusted for age and sex.

^b^HRs and 95% CI from Cox regression models adjusted for age and sex.

Longitudinally, no independent associations were found between any of the CAQ and PASE scores and rate of cognitive decline across all three cognitive domains and diagnostic groups, suggesting that at similar levels of atrophy, cognitive and physical activities do not modify (i.e. accelerate or slow down) cognitive decline (Supplementary Table 9A–D).

Assessing whether cognitive or physical activity modified the effect of temporoparietal atrophy on cross-sectional cognition, higher current CAQ and PASE were associated with a more negative effect of temporoparietal atrophy on baseline level of MMSE and executive function in the total sample. This reversed effect was mostly driven by the AD subgroup (Supplementary Table 10A–D). Investigating the moderating effect of activity on the association of atrophy with rate of cognitive decline, an interaction was observed only in the AD group. A higher level of current CAQ was associated with an attenuated effect of atrophy on rate of decline in EF (β_Std_ = 0.1, *P*_FDR_ < 0.05). This effect was observed also when adjusting for education and for *APOE-ɛ4* status, although it was not observed for MMSE or memory (Supplementary Table 11A–D).

### Associations with clinical progression and mortality

#### Cognitive and physical activities and clinical progression

**Figure 4:**
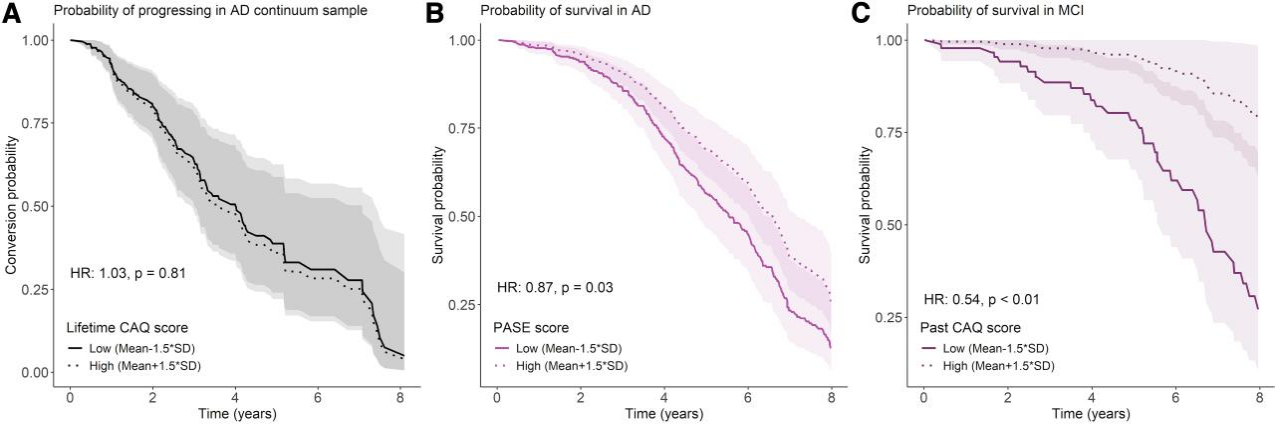
**Cox-model estimated survival curves.** (**A**) Progression in preclinical AD continuum participants by lifetime CAQ, (**B**) survival in AD individuals by PASE score and (**C**) survival in MCI individuals by past CAQ score. Survival probabilities were estimated using Cox regression models adjusted for age, sex and temporoparietal atrophy. Questionnaire scores were dichotomized with a median-split for plotting. High and low groups for illustrative purposes reflect ±1.5 SD from the mean. Note: The data and analyses depicted in this figure pertain only to participants within the amyloid-positive AD continuum sample.

Progression to MCI/dementia occurred in 29% of the SCD sample, and 43% of the MCI individuals converted to dementia during follow-up. Cox regression models in the predementia (i.e. combined SCD/MCI) group showed no association of any of the cognitive and physical activity questionnaire scores with risk of clinical progression, when controlling for the level of cortical atrophy (Table 4, Fig. 4A).

#### Cognitive and physical activities and mortality

A third of the total AD continuum sample died over the follow-up period [median (IQR) = 4.28 (2.57, 6.33)]: 9% of SCD, 21% of MCI and 42% of AD dementia patients. In the total sample, survival Cox models showed that higher cognitive and physical activities was associated with lower mortality rates, i.e. lifetime CAQ [HR (95% confidence interval, CI) = 0.87 (0.78–0.98), *P*_FDR_ < 0.05], current CAQ [HR (95% CI) = 0.81 (0.73–0.92), *P*_FDR_ < 0.001] and PASE [HR (95% CI) = 0.88 (0.79–0.99), *P*_FDR_ < 0.05]. The effect of lifetime [HR (95% CI) = 0.53 (0.35–0.8), *P*_FDR_ < 0.01] and past [HR (95% CI) = 0.54 (0.36–0.8), *P*_FDR_ < 0.01] CAQ on reduced mortality risk was mostly observed in individuals with MCI (Fig. 4B). The association of current CAQ [HR (95% CI) = 0.88 (0.77–1)] and PASE [HR (95% CI) = 0.87 (0.76–0.98)] levels with mortality was mostly driven by the AD dementia group, in which higher activity levels in individuals with similar atrophy relate to lower risk of death (Table 4, Fig. 4C). Comparable to the longitudinal cognition results described in the previous section, additionally adjusting the models for education and *APOE-ɛ4* status partially reduced the effect sizes (Supplementary Table 12A–C).

## Discussion

In this study, we employed two self-reported questionnaires to quantify and characterize two modifiable lifestyle factors, i.e. cognitive activity, defined in early-life, mid-life or concurrently at enrollment in the memory clinic, and physical activity, rated (only) at enrollment. Within the AD continuum, higher levels of both past and current cognitive activity, as well as current physical activity, were primarily associated with better baseline cognitive performance when controlling for temporoparietal brain atrophy. In other words, at similar amounts of AD-related brain atrophy, individuals engaging more often in these activities showed, on average, better cognitive performance, indicative of resilience. These protective effects were more pronounced in global cognition and executive functioning, and less so in the memory domain. Associations with global cognition were primarily driven by the AD dementia group, whereas the associations of cognitive activity with executive function were also observed in SCD and MCI. This pattern may suggest that lifestyle factors are more effective in preserving non-memory cognitive domains, whereas memory performance, often most compromised in individuals on the AD continuum, may present limited capacity for benefit. While the relationship between higher ‘current’ activity levels (i.e. measured at the first memory clinic visit) and higher concurrent cognitive performance may be partially attributable to reverse causality (i.e. more clinically impaired individuals may gradually engage less in cognitive/physical activities in their daily life), the observed effects with past cognitive activity suggest that early-life and mid-life engagement in mentally stimulating activities may play a role in building cognitive reserve. This reserve may provide a buffer against cognitive loss once AD manifests, allowing individuals to maintain better cognitive function for a longer period despite advancing pathology. Our findings align with previous literature showing a similar positive effect of early-life and adulthood cognitive activity on late-life cognition,^48^ suggesting a link between sustained intellectual engagement that requires mental effort and an increased efficiency and flexibility of neuronal circuits underlying cognition. Furthermore, our findings support evidence from a randomized controlled trial where cognitive and physical activities (combined with diet and vascular risk management) improved executive function, processing speed and global cognition, but not in memory, in elderly at risk of cognitive impairment.^49^

### Cognitive and physical activities and cognitive reserve

To examine resilience specifically in the context of AD, all longitudinal analyses were restricted to amyloid-positive individuals, optimizing biological homogeneity by reducing confounding from non-AD aetiologies. Importantly, our results indicate that the benefits of cognitive and physical activities across the AD continuum mainly pertained to a higher baseline cognitive performance rather than an attenuation of clinical progression. This is supported by the lack of associations with longitudinal cognitive decline or progression to MCI or dementia. In other words, past cognitive activity likely enhances premorbid cognitive performance (reflected by the intercept associations in our data) which, in turn, delays the onset of cognitive loss rather than modifying the rate of clinical progression (reflected by the lack of an association with the slope). These findings mirror our previous study on the same cohort that failed to observe a longitudinal association of educational attainment, the most commonly used proxy measure of cognitive reserve, with cognitive decline in prodromal AD stages.^50^ This is further supported by previous literature.^51-55^ Interestingly, we did not observe a paradoxical inverted association of cognitive/physical activity with an accelerated rate of decline in the later dementia stages, whereas this is consistently observed when using educational attainment as a predictor.

It is important to note that the difference in intercepts, but not in slopes, could also reflect what has been denoted as a ‘preserved differentiation’ of cognitive abilities, i.e. the differences observed in late-life cognition might simply reflect a persistence of functional differences that have existed since earlier life.^53,56^ While a proportion of the effect is indeed likely due to pre-disease differences in cognitive ability, this does not rule out the possibility of lifestyle factors having a beneficial causal effect on said pre-morbid levels earlier in life. Past research suggested a link between more stimulating environments in childhood and higher cognitive abilities later in life.^57-59^

### Cognitive and physical activities and mortality

In line with previous work on the same cohort in which other reserve proxies (i.e. educational attainment and total intracranial volume)^50^ were investigated, we observed a protective association of cognitive and physical activities with mortality. Current cognitive and physical activities were positively associated with a reduced risk of mortality in the overall sample, with this effect being most pronounced in the AD subgroup. However, due to the nature of these data and study design, it is impossible to separate a potential active benefit of these lifestyle factors from reverse causality effects, with the latter likely playing a more significant role in these results. Nonetheless, previous literature studying a population-based sample of individuals with AD dementia prospectively showed a prolonged survival of AD individuals that were more physically active prior to diagnosis.^60^ Similarly, a meta-analysis on the role of physical activity even in advanced dementia stages suggested that those engaging in higher activity decline at a lower rate compared to those who do not.^15^ Furthermore, in our results, lifetime and past cognitive activity provided a (comparably larger) survival benefit for individuals with MCI, although not for those with SCD or dementia. While this may reflect broader health-related behaviours or earlier-stage intervention effects, we cannot rule out the role of unmeasured confounders, recall bias or noise. Literature on the relationship between early- and mid-life cognitively stimulating activities and mortality outcomes is scarce, and therefore, the potential mechanisms remain elusive. Nonetheless, modifiable lifestyle factors are increasingly recognized as key targets for intervention to promote cognitive health, and potentially longevity, in the face of AD pathology.^61,62^

### Cognitive and physical activities in a memory clinic cohort

Descriptive analyses of the full memory clinic sample showed that diagnostic groups differed mainly in current cognitive and physical activity levels, likely reflecting clinical severity rather than disease-specific aetiologies. Females had higher lifetime, past and current cognitive activity scores, while males had higher physical activity scores. Examining the association with atrophy, physical (but not cognitive) activity showed a modest positive association with higher whole-brain cortical thickness. All questionnaire summary scores were positively associated with educational attainment and cognitive performance, underscoring the connection between cognitive reserve proxies and better outcomes across neurodegenerative diseases. Investigating different cognitive domains, current cognitive activity was associated with higher scores on all three outcomes, i.e. global cognition, memory and executive function, likely reflecting partial reverse causality. Past cognitive activity, however, was related to better global cognition and executive function, but not to memory. Similarly, within the AD continuum, past and current cognitive activity and current physical activity were primarily associated with better baseline cognitive performance when controlling for temporoparietal brain atrophy, particularly in global cognition and executive functioning. This finding is supported by extensive literature linking cognitive reserve to superior functioning specifically of cognitive abilities that support efficient processing of information in the brain.^63-66^ Furthermore, some interventional studies aimed at improving cognition through physical activity showed benefits on executive functioning^67,68^ in individuals with MCI or dementia; however, interventions with cognitive training failed to show a significant benefit in these patients.^69^

### Strengths and limitations

This study has several strengths. Firstly, the large memory clinic cohort with consecutive enrollment provides a unique sample for investigating the complex relationships between cognitive and physical activities, dementia-related brain atrophy and cognition. Unlike studies focusing on broadly defined dementia groups or narrow diagnostic subsets, our cohort includes a wide range of clinical and aetiological diagnoses. A further asset is the comprehensive assessment of both cognitive and physical activities across the lifespan using questionnaire-based measures in one sample, allowing for a more direct and straightforward comparison between the two lifestyle factors. There are also several limitations. Firstly, self-reported measures of cognitive and physical activities introduce potential recall bias and subjective reporting, particularly for early-life items and in individuals with advanced cognitive impairment. While caregivers often assisted with survey completion, we lacked systematic data to account for proxy effects in our analyses. Future developments in digital measures will likely facilitate the objective assessment of cognitive and physical activity measures. Secondly, the memory clinic setting led to variability in missing data across variables and time points. Longitudinal cognitive assessments were not uniformly collected, and dropout rates were higher among the more impaired individuals. Thirdly, we lacked socioeconomic status information and cardiovascular health data for the full sample. Given established links between cognitive activity, education, socioeconomic status and cardiovascular risk, these omissions may have influenced our findings. Furthermore, the ADC cohort, composed mainly of highly educated, mostly Caucasian individuals with higher socioeconomic status, may not represent the average Dutch memory clinic patient. Future studies should include more comprehensive assessment of these variables. Similarly, additional pathological markers (e.g. tau and cerebrovascular disease) that likely influence clinical outcomes are key to include in future studies to disentangle the relative contributions of co-pathologies and better characterize the mechanisms underlying cognitive and brain resilience. Fourthly, as an observational study, causal inferences between lifestyle activities and cognitive outcomes cannot be definitively made. Experimental designs are essential for determining optimal types, frequencies and durations of cognitive and physical activities for maximizing brain health and promoting successful ageing amidst AD pathology.

## Conclusion

Engaging in cognitively stimulating activities during early- and mid-life may provide late-life cognitive benefits that persist even when AD manifests. While associations between current cognitive and physical activities and better cognitive performance could partly reflect reverse causality, the effects of past cognitive activity underscore the value of lifelong cognitive engagement to promote cognitive health in old age and neurodegeneration.

## Supplementary Material

fcaf318_Supplementary_Data

## Data Availability

Anonymized data will be shared by request from a qualified academic investigator for the sole purpose of replicating procedures and results presented in the article if data transfer is in agreement with relevant legislation on the general data protection regulation and decisions and by the relevant Ethical Review Boards, which should be regulated in a material transfer agreement. The underlying code for this study is accessible at accessible at https://github.com/OssenKoppeLab.
