# Supplementary material for: Cognitive and physical activities are associated with cognitive resilience in a memory clinic cohort
Source: Brain Commun. 2025 Sep 6;7(5):fcaf318. doi: 10.1093/braincomms/fcaf318 (PMC12516313; doi:10.1093/braincomms/fcaf318)
Supplement: fcaf318_Supplementary_Data [file fcaf318_supplementary_data.pdf]

Supplement of *Cognitive and physical activity are associated with cognitive resilience in a memory-clinic cohort*

Supplementary Table 1. Missing values across cohort and within diagnostic groups

Supplementary Table 2. Characteristics Amsterdam Dementia Cohort – summary statistics calculated with multiple imputed datasets

Supplementary Table 3. Characteristics Alzheimer's Disease Cohort - summary statistics calculated with Complete Case data

Supplementary Table 4. Models tested in part 1 – Descriptives

Supplementary Table 5. Differences in cognitive and physical activity across diagnostic groups

Supplementary Figure 1. Cognition over time

Supplementary Table 6. Longitudinal models tested in part 2 – Cognitive/physical activity and cognitive resilience

Supplementary Table 7. Linear mixed-effects models – random intercepts and slopes

Supplementary Table 8a. Cross-sectional main effects on cognition independent of temporoparietal cortical thickness, adjusted for education, operationalized as tertiles

Supplementary Table 8b. Cross-sectional main effects on cognition independent of temporoparietal cortical thickness, adjusted for education, operationalized as (Verhage-transformed) years of education.

Supplementary Table 8c. Cross-sectional main effects on cognition independent of temporoparietal cortical thickness, adjusted for education, operationalized as tertiles, and for APOE-ε4 carriership status

Supplementary Table 9a. Longitudinal main effects on cognition independent of temporoparietal cortical thickness

Supplementary Table 9b. Longitudinal main effects on cognition independent of temporoparietal cortical thickness, adjusted for education, operationalized as tertiles

Supplementary Table 9c. Longitudinal main effects on cognition independent of temporoparietal cortical thickness, adjusted for education, operationalized as (Verhage-transformed) years of education.

Supplementary Table 9d. Longitudinal main effects on cognition independent of temporoparietal cortical thickness, adjusted for education, operationalized as tertiles, and for APOE-ε4 carriership status

Supplementary Table 10a. Two-way interaction effect of cognitive/physical activity with temporoparietal cortical thickness on baseline cognition

Supplementary Table 10b. Two-way interaction effect of cognitive/physical activity with temporoparietal cortical thickness on baseline cognition, adjusted for education, operationalized as tertiles

Supplementary Table 10c. Two-way interaction effect of cognitive/physical activity with temporoparietal cortical thickness on baseline cognition, adjusted for education, operationalized as (Verhage-transformed) years of education.

Supplementary Table 10d. Two-way interaction effect of cognitive/physical activity with temporoparietal cortical thickness on baseline cognition, adjusted for education, operationalized as tertiles, and for APOE-ε4 carriership status

Supplementary Table 11a. Three-way interaction effect of cognitive/physical activity with temporoparietal cortical thickness on longitudinal rate of decline

Supplementary Table 11b. Three-way interaction effect of cognitive/physical activity with temporoparietal cortical thickness on longitudinal rate of decline, adjusted for education, operationalized as tertiles

Supplementary Table 11c. Three-way interaction effect of cognitive/physical activity with temporoparietal cortical thickness on longitudinal rate of decline, adjusted for education, operationalized as (Verhage-transformed) years of education.

Supplementary Table 11d. Three-way interaction effect of cognitive/physical activity with temporoparietal cortical thickness on longitudinal rate of decline, adjusted for education, operationalized as tertiles, and for APOE-ε4 carriership status

Supplementary Table 12a. Risk of progression and of mortality, adjusted for education operationalized as tertiles

Supplementary Table 12b. Risk of progression and of mortality, adjusted for education, operationalized as (Verhage-transformed) years of education.

Supplementary Table 12c. Risk of progression and of mortality, adjusted for education, operationalized as tertiles, and for APOE-ε4 carriership status

Supplementary Table 1. Missing values across cohort and within diagnostic groups

**Characteristic**	**N**	**Total** N = 4033	**SCD** N = 988	**MCI** N = 524	**AD** N = 1208	**PPA** N = 84	**FTD** N = 198	**DLB** N = 154	**CBS/PSP** N = 78	**VaD** N = 77	**Dem other** N = 65	**Neuro other** N = 186	**Psych** N = 471
**Age**	4,033	0.0	0.0	0.0	0.0	0.0	0.0	0.0	0.0	0.0	0.0	0.0	0.0
**Sex**	4,033	0	0	0	0	0	0	0	0	0	0	0	0
**Education**	4,018	0.4	0.7	0.2	<0.1	0	1.5	0	1.3	0	0	0	0.4
**APOE e4 status**	3,925	2.7	3.6	2.9	2.3	0	3.5	3.2	3.8	0	0	2.2	2.1
**Amyloid status**	3,241	20	24	12	12	17	21	27	32	22	20	31	28
**MMSE**	3,947	2.1	0.8	0.2	2.7	2.4	4.5	2.6	5.1	3.9	4.6	1.6	3.4
**Memory**	3,709	8.0	2.6	2.9	10.9	14.3	13.1	11.7	5.1	14.3	12.3	10.2	11.3
**Executive function**	3,695	8.4	2.3	2.5	12.2	9.5	16.7	12.3	9.0	22.1	12.3	9.1	9.8
**Lifetime CAQ**	3,515	12.8	9.6	11.6	13.1	11.9	13.6	13.0	12.8	11.7	29.2	17.2	16.3
**Past CAQ**	3,553	11.9	8.4	11.3	12.0	11.9	13.6	12.3	11.5	11.7	27.7	14.0	15.9
**Current CAQ**	3,879	3.8	2.9	2.7	3.9	1.2	4.0	3.2	5.1	2.6	9.2	5.9	5.7
**PASE score**	3,338	17.2	17.3	15.8	18.1	21.4	20.2	18.2	17.9	18.2	20.0	15.1	14.2
**Wholebrain thickness**	2,885	28.5	18.0	19.7	33.9	36.9	41.4	27.3	43.6	39.0	43.1	38.2	29.5

% of missing values within each group are presented (except for the “N” column that represents the number of individuals with available data for each variable).

SCD = Subjective cognitive decline, MCI = Mild cognitive impairment, AD = Alzheimer's disease dementia, PPA = Primary progressive aphasia, FTD = Frontotemporal neurodegeneration dementia, DLB = Lewy Body disease, CBS/PSP = Corticobasal syndrome / Progressive supranuclear palsy, VaD = Vascular dementia, Dem other = other dementias, Neuro other = other neurological disorders, Psych = Psychiatry

Supplementary Table 2. Characteristics Amsterdam Dementia Cohort – summary statistics calculated with multiple imputed datasets

Variable	N	Total	SCD	MCI	AD	PPA	FTD	DLB	CBS/PSP	VaD	Dem other	Neuro other	Psych
**N**	4,033	4033	988	524	1208	84	198	154	78	77	65	186	471
**Age**	4,033	63.4 ± 8.4	61.1 ± 8.4	65.7 ± 7.2	65.4 ± 7.6	66.3 ± 7.9	63.6 ± 7.7	68.6 ± 5.3	66.8 ± 6.0	67.8 ± 8.2	64.3 ± 9.8	61.8 ± 8.7	57.9 ± 8.9
**Sex, N females (%)**	4,033	1,719 (43)	408 (41)	194 (37)	637 (53)	42 (50)	90 (45)	33 (21)	30 (38)	28 (36)	23 (35)	62 (33)	172 (37)
**Education, N (%)**	4,033												
Low		1,228 (30)	194 (20)	144 (27)	407 (34)	24 (29)	72 (36)	43 (28)	32 (41)	32 (42)	23 (35)	70 (38)	187 (40)
Medium		1,132 (28)	263 (27)	139 (27)	346 (29)	25 (30)	59 (30)	38 (25)	21 (27)	25 (32)	24 (37)	57 (31)	135 (29)
High		1,673 (41)	531 (54)	241 (46)	455 (38)	35 (42)	67 (34)	73 (47)	25 (32)	20 (26)	18 (28)	59 (32)	149 (32)
**APOE e4, N positive (%)**	4,033	1,931 (48)	367 (37)	292 (56)	827 (68)	25 (30)	68 (34)	79 (51)	27 (35)	31 (40)	13 (20)	52 (28)	150 (32)
**Amyloid status**	n.a.	n.a.	n.a.	n.a.	n.a.	n.a.	n.a.	n.a.	n.a.	n.a.	n.a.	n.a.	n.a.
**MMSE**	4,033	24.5 ± 5.0	28.2 ± 1.7	26.6 ± 2.3	20.4 ± 5.3	24.5 ± 4.8	23.1 ± 5.3	22.9 ± 4.4	24.1 ± 3.8	22.9 ± 4.6	23.1 ± 5.3	26.2 ± 3.5	25.8 ± 3.8
**Memory**	4,033	-2.1 ± 2.3	-0.2 ± 0.9	-2.0 ± 1.5	-4.3 ± 2.0	-1.7 ± 1.7	-2.1 ± 1.8	-2.9 ± 1.8	-1.4 ± 1.5	-2.8 ± 1.9	-2.7 ± 2.1	-1.4 ± 1.6	-1.1 ± 1.5
**Executive function**	4,033	-1.2 ± 1.4	-0.1 ± 0.9	-0.8 ± 0.9	-2.0 ± 1.3	-1.4 ± 1.3	-1.6 ± 1.3	-2.1 ± 1.1	-2.9 ± 1.3	-2.2 ± 1.3	-2.5 ± 1.6	-1.2 ± 1.3	-1.1 ± 1.2
**Lifetime CAQ**	4,033	2.59 ± 0.64	2.71 ± 0.63	2.65 ± 0.62	2.59 ± 0.62	2.64 ± 0.66	2.50 ± 0.68	2.60 ± 0.58	2.48 ± 0.64	2.36 ± 0.71	2.35 ± 0.67	2.56 ± 0.63	2.39 ± 0.67
**Past CAQ**	4,033	2.56 ± 0.67	2.65 ± 0.66	2.57 ± 0.65	2.58 ± 0.66	2.59 ± 0.74	2.49 ± 0.70	2.59 ± 0.62	2.43 ± 0.69	2.33 ± 0.73	2.36 ± 0.72	2.53 ± 0.66	2.40 ± 0.71
**Current CAQ**	4,033	2.71 ± 0.83	2.95 ± 0.78	2.95 ± 0.76	2.65 ± 0.80	2.84 ± 0.74	2.52 ± 0.87	2.64 ± 0.75	2.64 ± 0.79	2.47 ± 0.91	2.33 ± 0.79	2.67 ± 0.83	2.34 ± 0.85
**PASE score**	4,033	138.2 ± 84.0	157.7 ± 85.2	147.9 ± 81.2	139.8 ± 78.8	172.4 ± 85.7	130.4 ± 94.9	133.4 ± 91.7	99.8 ± 59.2	87.9 ± 82.1	83.7 ± 68.9	112.2 ± 67.9	113.6 ± 82.7
**Wholebrain thickness**	n.a.	n.a.	n.a.	n.a.	n.a.	n.a.	n.a.	n.a.	n.a.	n.a.	n.a.	n.a.	n.a.

Mean +/- Standard Deviation, unless specified otherwise.
Note that Amyloid status, AD signature thickness and Wholebrain thickness variables were not imputed (given the higher rate of missing values).
SCD = Subjective cognitive decline, MCI = Mild cognitive impairment, AD = Alzheimer's disease dementia, PPA = Primary progressive aphasia, FTD = Frontotemporal neurodegeneration dementia, DLB = Lewy Body disease, CBD/PSD = Corticobasal Degeneration / Progressive supranuclear palsy, VaD = Vascular dementia, Dem other = other dementias, Neuro other = other neurological disorders, Psych = Psychiatry

Supplementary Table 3. Characteristics Alzheimer's Disease Cohort - summary statistics calculated with Complete Case data

Variable	N	Total	SCD	MCI	AD	PPA	FTD	DLB	CBS/PSP	VaD	Dem other	Neuro other	Psych
**N**	4,033	4033	988	524	1208	84	198	154	78	77	65	186	471
**Age**	4,033	63.4 ± 8.4	61.1 ± 8.4	65.7 ± 7.2	65.4 ± 7.6	66.3 ± 7.9	63.6 ± 7.7	68.6 ± 5.3	66.8 ± 6.0	67.8 ± 8.2	64.3 ± 9.8	61.8 ± 8.7	57.9 ± 8.9
**Sex, N females (%)**	4,033	1,719 (43)	408 (41)	194 (37)	637 (53)	42 (50)	90 (45)	33 (21)	30 (38)	28 (36)	23 (35)	62 (33)	172 (37)
**Education, N (%)**	4,018												
Low		1,225 (30)	194 (20)	143 (27)	407 (34)	24 (29)	71 (36)	43 (28)	32 (42)	32 (42)	23 (35)	70 (38)	186 (40)
Medium		1,132 (28)	263 (27)	139 (27)	346 (29)	25 (30)	59 (30)	38 (25)	21 (27)	25 (32)	24 (37)	57 (31)	135 (29)
High		1,661 (41)	524 (53)	241 (46)	454 (38)	35 (42)	65 (33)	73 (47)	24 (31)	20 (26)	18 (28)	59 (32)	148 (32)
**APOE e4, N positive (%)**	3,925	1,883 (48)	361 (38)	283 (56)	801 (68)	25 (30)	66 (35)	75 (50)	27 (36)	31 (40)	13 (20)	52 (29)	149 (32)
**Amyloid status, N positive (%)**	3,241	1,730 (53)	174 (23)	264 (57)	1,014 (95)	25 (36)	36 (23)	69 (62)	14 (26)	23 (38)	23 (44)	30 (23)	58 (17)
**MMSE**	3,947	24.5 ± 5.0	28.2 ± 1.7	26.6 ± 2.3	20.4 ± 5.3	24.6 ± 4.8	23.2 ± 5.3	22.9 ± 4.4	24.1 ± 3.9	22.9 ± 4.6	23.2 ± 5.3	26.2 ± 3.6	25.8 ± 3.8
**Memory**	3,709	-2.0 ± 2.2	-0.1 ± 0.9	-2.0 ± 1.5	-4.2 ± 2.0	-1.6 ± 1.6	-2.0 ± 1.7	-2.8 ± 1.8	-1.4 ± 1.5	-2.7 ± 1.9	-2.5 ± 2.0	-1.3 ± 1.6	-1.0 ± 1.4
**Executive function**	3,695	-1.2 ± 1.4	-0.1 ± 0.9	-0.8 ± 0.9	-1.9 ± 1.3	-1.3 ± 1.3	-1.5 ± 1.3	-2.0 ± 1.1	-2.9 ± 1.3	-2.2 ± 1.3	-2.5 ± 1.7	-1.1 ± 1.3	-1.0 ± 1.1
**Lifetime CAQ**	3,515	2.6 ± 0.6	2.7 ± 0.6	2.7 ± 0.6	2.6 ± 0.6	2.6 ± 0.7	2.5 ± 0.7	2.6 ± 0.6	2.5 ± 0.6	2.4 ± 0.7	2.2 ± 0.6	2.6 ± 0.6	2.4 ± 0.7
**Past CAQ**	3,553	2.6 ± 0.7	2.7 ± 0.7	2.6 ± 0.6	2.6 ± 0.7	2.6 ± 0.8	2.5 ± 0.7	2.6 ± 0.6	2.4 ± 0.7	2.3 ± 0.7	2.2 ± 0.6	2.5 ± 0.7	2.4 ± 0.7
**Current CAQ**	3,879	2.7 ± 0.8	3.0 ± 0.8	3.0 ± 0.8	2.6 ± 0.8	2.8 ± 0.7	2.5 ± 0.9	2.6 ± 0.8	2.7 ± 0.8	2.5 ± 0.9	2.3 ± 0.8	2.7 ± 0.8	2.3 ± 0.9
**PASE score**	3,338	134.2 ± 81.4	153.7 ± 81.8	144.3 ± 79.6	135.0 ± 77.3	170.7 ± 89.1	128.1 ± 87.7	125.2 ± 91.2	96.9 ± 52.2	77.9 ± 75.4	84.1 ± 70.6	111.5 ± 68.8	111.3 ± 78.9
**Wholebrain thickness**	2,885	2.3 ± 0.1	2.4 ± 0.1	2.3 ± 0.1	2.3 ± 0.1	2.3 ± 0.1	2.3 ± 0.1	2.3 ± 0.1	2.3 ± 0.1	2.3 ± 0.1	2.3 ± 0.1	2.4 ± 0.1	2.4 ± 0.1

Mean +/- Standard Deviation, unless specified otherwise.
Summary statistics presented here were calculated based on a complete-case analyses (with varying number of available individual data points N per variable). Summary statistics calculated within a multiple imputation framework are shown in Supplementary Table 2.
SCD = Subjective cognitive decline, MCI = Mild cognitive impairment, AD = Alzheimer's disease dementia, PPA = Primary progressive aphasia, FTD = Frontotemporal neurodegeneration dementia, DLB = Dementia with Lewy Bodies, CBS/PSP = Corticobasal Degeneration / Progressive supranuclear palsy, VaD = Vascular dementia, Dem other = other dementias, Neuro other = other neurological disorders, Psych = Psychiatry

Supplementary Table 4. Models tested in part 1 – Descriptives

**Predictor of interest**	**Model**
Diagnosis	CAQ score / PASE score ~ **diagnosis** + age + sex + education
Sex	CAQ score / PASE score ~ **sex** + age + education + diagnosis
Education	CAQ score / PASE score ~ **education** + age + sex + diagnosis
APOE e4 status	CAQ score / PASE score ~ **APOE e4 status** + age + sex + education + diagnosis
Age	CAQ score / PASE score ~ **age** + sex + education + diagnosis
MMSE	CAQ score / PASE score ~ **MMSE** + age + sex + education + diagnosis
Memory	CAQ score / PASE score ~ **Memory** + age + sex + education + diagnosis
Executive function	CAQ score / PASE score ~ **Executive function** + age + sex + education + diagnosis
Lifetime CAQ	CAQ score / PASE score ~ **lifetime CAQ** + age + sex + education + diagnosis
Past CAQ	CAQ score / PASE score ~ **past CAQ** + age + sex + education + diagnosis<br>CAQ score / Lifetime CAQ ~ **past CAQ** + age + sex + education + diagnosis
Current CAQ	CAQ score / PASE score ~ **current CAQ** + age + sex + education + diagnosis<br>CAQ score / Lifetime CAQ ~ **current CAQ** + age + sex + education + diagnosis<br>CAQ score / Past CAQ ~ **current CAQ** + age + sex + education + diagnosis
Wholebrain thickness	CAQ score / PASE score ~ **Wholebrain thickness** + age + education + diagnosis

Supplementary Table 5. Differences in cognitive and physical activity across diagnostic groups

	CAQ lifetime score			CAQ past score			CAQ current score			PASE score		
contrast	Estimate (SD)	Std. Estimate (SD)	p-value	Estimate (SD)	Std. Estimate (SD)	p-value	Estimate (SD)	Std. Estimate (SD)	p-value	Estimate (SD)	Std. Estimate (SD)	p-value
SCD - MCI	0.03 (0.03)	0.04 (0.05)	0.999	0.02 (0.03)	0.03 (0.05)	1.000	0.04 (0.04)	0.04 (0.05)	0.998	3.28 (4.52)	0.04 (0.06)	1.000
SCD - AD	0.05 (0.02)	0.08 (0.04)	0.578	-0.01 (0.03)	-0.02 (0.04)	1.000	0.31 (0.03)	0.37 (0.04)	**<0.001**	8.76 (3.67)	0.11 (0.04)	0.373
SCD - PPA	0.04 (0.06)	0.06 (0.1)	1.000	0.01 (0.07)	0.02 (0.1)	1.000	0.16 (0.08)	0.19 (0.1)	0.719	-24.45 (9.37)	-0.3 (0.11)	0.242
SCD - FTD	0.11 (0.04)	0.17 (0.07)	0.362	0.04 (0.05)	0.06 (0.07)	0.999	0.36 (0.06)	0.44 (0.07)	**<0.001**	21.51 (6.48)	0.26 (0.08)	**0.035**
SCD - DLB	0.07 (0.05)	0.1 (0.08)	0.963	-0.01 (0.05)	-0.02 (0.08)	1.000	0.38 (0.07)	0.46 (0.08)	**<0.001**	16.53 (7.23)	0.2 (0.09)	0.433
SCD - (CBS/PSP)	0.11 (0.07)	0.17 (0.1)	0.883	0.07 (0.07)	0.1 (0.11)	0.998	0.28 (0.09)	0.34 (0.11)	0.067	48.5 (9.86)	0.59 (0.12)	**<0.001**
SCD - VaD	0.2 (0.07)	0.32 (0.1)	0.084	0.14 (0.07)	0.21 (0.11)	0.654	0.45 (0.09)	0.54 (0.11)	**<0.001**	59.25 (9.76)	0.72 (0.12)	**<0.001**
SCD - Dem other	0.22 (0.07)	0.34 (0.11)	0.103	0.14 (0.08)	0.2 (0.12)	0.815	0.55 (0.1)	0.66 (0.12)	**<0.001**	69.26 (10.43)	0.84 (0.13)	**<0.001**
SCD - Neuro other	0 (0.04)	0 (0.07)	1.000	-0.04 (0.05)	-0.05 (0.07)	1.000	0.16 (0.06)	0.19 (0.07)	0.224	44.3 (6.54)	0.54 (0.08)	**<0.001**
SCD - Psych	0.15 (0.03)	0.23 (0.05)	**<0.001**	0.08 (0.03)	0.11 (0.05)	0.458	0.41 (0.04)	0.5 (0.05)	**<0.001**	47.6 (4.67)	0.58 (0.06)	**<0.001**
MCI - AD	0.03 (0.03)	0.04 (0.05)	0.999	-0.03 (0.03)	-0.05 (0.05)	0.991	0.27 (0.04)	0.33 (0.05)	**<0.001**	5.48 (4.31)	0.07 (0.05)	0.969
MCI - PPA	0.01 (0.07)	0.02 (0.1)	1.000	-0.01 (0.07)	-0.02 (0.1)	1.000	0.12 (0.09)	0.15 (0.11)	0.942	-27.73 (9.63)	-0.34 (0.12)	0.132
MCI - FTD	0.08 (0.05)	0.12 (0.07)	0.841	0.02 (0.05)	0.03 (0.07)	1.000	0.33 (0.06)	0.4 (0.08)	**<0.001**	18.23 (6.9)	0.22 (0.08)	0.211
MCI - DLB	0.04 (0.05)	0.06 (0.08)	1.000	-0.03 (0.06)	-0.05 (0.08)	1.000	0.34 (0.07)	0.42 (0.08)	**<0.001**	13.25 (7.54)	0.16 (0.09)	0.789
MCI - (CBS/PSP)	0.08 (0.07)	0.12 (0.11)	0.985	0.04 (0.07)	0.06 (0.11)	1.000	0.24 (0.09)	0.29 (0.11)	0.225	45.22 (10.11)	0.55 (0.12)	**<0.001**
MCI - VaD	0.18 (0.07)	0.27 (0.11)	0.260	0.12 (0.07)	0.18 (0.11)	0.869	0.41 (0.09)	0.5 (0.11)	**<0.001**	55.97 (9.99)	0.69 (0.12)	**<0.001**
MCI - Dem other	0.19 (0.07)	0.29 (0.12)	0.278	0.11 (0.08)	0.17 (0.12)	0.945	0.51 (0.1)	0.62 (0.12)	**<0.001**	65.97 (10.68)	0.81 (0.13)	**<0.001**
MCI - Neuro other	-0.02 (0.05)	-0.04 (0.07)	1.000	-0.06 (0.05)	-0.09 (0.08)	0.987	0.12 (0.06)	0.15 (0.08)	0.707	41.02 (7)	0.5 (0.09)	**<0.001**
MCI - Psych	0.12 (0.04)	0.19 (0.06)	**0.049**	0.05 (0.04)	0.08 (0.06)	0.951	0.38 (0.05)	0.46 (0.06)	**<0.001**	44.31 (5.36)	0.54 (0.07)	**<0.001**
AD - PPA	-0.01 (0.06)	-0.02 (0.1)	1.000	0.02 (0.07)	0.03 (0.1)	1.000	-0.15 (0.08)	-0.18 (0.1)	0.807	-33.21 (9.25)	-0.4 (0.11)	**0.015**
AD - FTD	0.05 (0.04)	0.08 (0.07)	0.977	0.05 (0.05)	0.08 (0.07)	0.990	0.06 (0.06)	0.07 (0.07)	0.996	12.75 (6.33)	0.16 (0.08)	0.628
AD - DLB	0.01 (0.05)	0.02 (0.08)	1.000	0 (0.05)	0 (0.08)	1.000	0.07 (0.06)	0.09 (0.08)	0.989	7.76 (7.06)	0.1 (0.09)	0.990
AD - (CBS/PSP)	0.05 (0.07)	0.09 (0.1)	0.999	0.08 (0.07)	0.11 (0.1)	0.991	-0.03 (0.09)	-0.04 (0.11)	1.000	39.74 (9.72)	0.48 (0.12)	**0.002**
AD - VaD	0.15 (0.07)	0.23 (0.1)	0.440	0.15 (0.07)	0.23 (0.1)	0.514	0.14 (0.09)	0.17 (0.11)	0.888	50.49 (9.6)	0.62 (0.12)	**<0.001**
AD - Dem other	0.16 (0.07)	0.26 (0.11)	0.453	0.15 (0.08)	0.22 (0.11)	0.716	0.24 (0.1)	0.29 (0.12)	0.311	60.49 (10.33)	0.74 (0.13)	**<0.001**
AD - Neuro other	-0.05 (0.04)	-0.08 (0.07)	0.989	-0.02 (0.05)	-0.04 (0.07)	1.000	-0.15 (0.06)	-0.18 (0.07)	0.311	35.54 (6.46)	0.43 (0.08)	**<0.001**
AD - Psych	0.09 (0.03)	0.15 (0.05)	0.114	0.09 (0.03)	0.13 (0.05)	0.239	0.11 (0.04)	0.13 (0.05)	0.304	38.83 (4.63)	0.47 (0.06)	**<0.001**
PPA - FTD	0.07 (0.07)	0.1 (0.11)	0.998	0.03 (0.08)	0.04 (0.12)	1.000	0.2 (0.1)	0.25 (0.12)	0.575	45.96 (10.68)	0.56 (0.13)	**0.001**

PPA - DLB	0.03 (0.08)	0.04 (0.12)	1.000	-0.02 (0.08)	-0.03 (0.12)	1.000	0.22 (0.1)	0.27 (0.12)	0.526	40.98 (11.15)	0.5 (0.14)	**0.010**
PPA - (CBS/PSP)	0.07 (0.09)	0.1 (0.14)	1.000	0.05 (0.09)	0.08 (0.14)	1.000	0.12 (0.12)	0.14 (0.14)	0.996	72.95 (12.93)	0.89 (0.16)	**<0.001**
PPA - VaD	0.16 (0.09)	0.25 (0.14)	0.758	0.13 (0.09)	0.19 (0.14)	0.950	0.29 (0.12)	0.35 (0.14)	0.343	83.7 (12.89)	1.02 (0.16)	**<0.001**
PPA - Dem other	0.18 (0.09)	0.27 (0.14)	0.730	0.13 (0.1)	0.19 (0.15)	0.976	0.39 (0.12)	0.47 (0.15)	0.068	93.71 (13.5)	1.14 (0.16)	**<0.001**
PPA - Neuro other	-0.04 (0.07)	-0.06 (0.11)	1.000	-0.05 (0.08)	-0.07 (0.12)	1.000	0 (0.1)	0 (0.12)	1.000	68.75 (10.77)	0.84 (0.13)	**<0.001**
PPA - Psych	0.1 (0.07)	0.16 (0.1)	0.898	0.07 (0.07)	0.1 (0.11)	0.998	0.25 (0.09)	0.31 (0.11)	0.142	72.05 (9.82)	0.88 (0.12)	**<0.001**
FTD - DLB	-0.04 (0.06)	-0.06 (0.09)	1.000	-0.05 (0.07)	-0.08 (0.1)	0.999	0.02 (0.08)	0.02 (0.1)	1.000	-4.99 (8.85)	-0.06 (0.11)	1.000
FTD - (CBS/PSP)	0 (0.08)	0 (0.12)	1.000	0.03 (0.08)	0.04 (0.12)	1.000	-0.09 (0.1)	-0.11 (0.12)	0.999	26.99 (11.07)	0.33 (0.13)	0.342
FTD - VaD	0.1 (0.08)	0.15 (0.12)	0.971	0.1 (0.08)	0.15 (0.12)	0.974	0.08 (0.1)	0.1 (0.12)	0.999	37.74 (10.94)	0.46 (0.13)	**0.023**
FTD - Dem other	0.11 (0.08)	0.17 (0.13)	0.958	0.1 (0.09)	0.14 (0.13)	0.990	0.18 (0.11)	0.22 (0.13)	0.839	47.74 (11.64)	0.58 (0.14)	**0.002**
FTD - Neuro other	-0.1 (0.06)	-0.16 (0.09)	0.770	-0.08 (0.06)	-0.11 (0.09)	0.978	-0.21 (0.08)	-0.25 (0.09)	0.206	22.79 (8.37)	0.28 (0.1)	0.195
FTD - Psych	0.04 (0.05)	0.06 (0.07)	0.999	0.04 (0.05)	0.06 (0.08)	1.000	0.05 (0.06)	0.06 (0.08)	1.000	26.08 (7.02)	0.32 (0.09)	**0.009**
DLB - (CBS/PSP)	0.04 (0.08)	0.06 (0.12)	1.000	0.08 (0.08)	0.12 (0.12)	0.998	-0.1 (0.1)	-0.13 (0.13)	0.996	31.98 (11.48)	0.39 (0.14)	0.164
DLB - VaD	0.14 (0.08)	0.21 (0.12)	0.813	0.15 (0.08)	0.23 (0.12)	0.755	0.07 (0.1)	0.08 (0.13)	1.000	42.72 (11.39)	0.52 (0.14)	**0.008**
DLB - Dem other	0.15 (0.08)	0.23 (0.13)	0.787	0.15 (0.09)	0.22 (0.13)	0.864	0.17 (0.11)	0.2 (0.14)	0.922	52.73 (12.05)	0.64 (0.15)	**0.001**
DLB - Neuro other	-0.06 (0.06)	-0.1 (0.1)	0.994	-0.02 (0.07)	-0.04 (0.1)	1.000	-0.22 (0.08)	-0.27 (0.1)	0.205	27.77 (8.94)	0.34 (0.11)	0.073
DLB - Psych	0.08 (0.05)	0.12 (0.08)	0.928	0.09 (0.06)	0.13 (0.09)	0.899	0.03 (0.07)	0.04 (0.09)	1.000	31.07 (7.81)	0.38 (0.1)	**0.003**
(CBS/PSP) - VaD	0.1 (0.09)	0.15 (0.14)	0.993	0.08 (0.1)	0.11 (0.14)	0.999	0.17 (0.12)	0.2 (0.14)	0.946	10.75 (13.19)	0.13 (0.16)	0.999
(CBS/PSP) - Dem other	0.11 (0.1)	0.17 (0.15)	0.988	0.07 (0.1)	0.1 (0.15)	1.000	0.27 (0.13)	0.33 (0.15)	0.551	20.75 (13.79)	0.25 (0.17)	0.917
(CBS/PSP) - Neuro other	-0.1 (0.08)	-0.16 (0.12)	0.953	-0.1 (0.08)	-0.15 (0.12)	0.976	-0.12 (0.1)	-0.14 (0.12)	0.987	-4.2 (11.14)	-0.05 (0.14)	1.000
(CBS/PSP) - Psych	0.04 (0.07)	0.06 (0.11)	1.000	0.01 (0.07)	0.02 (0.11)	1.000	0.14 (0.09)	0.17 (0.11)	0.928	-0.91 (10.23)	-0.01 (0.12)	1.000
VaD - Dem other	0.01 (0.09)	0.02 (0.15)	1.000	-0.01 (0.1)	-0.01 (0.15)	1.000	0.1 (0.13)	0.12 (0.15)	0.999	10 (13.69)	0.12 (0.17)	1.000
VaD - Neuro other	-0.2 (0.08)	-0.31 (0.12)	0.225	-0.18 (0.08)	-0.26 (0.12)	0.509	-0.29 (0.1)	-0.35 (0.12)	0.145	-14.95 (11.09)	-0.19 (0.13)	0.955
VaD - Psych	-0.06 (0.07)	-0.09 (0.11)	0.999	-0.06 (0.07)	-0.1 (0.11)	0.999	-0.03 (0.09)	-0.04 (0.11)	1.000	-11.66 (10.17)	-0.14 (0.12)	0.986
Dem other - Neuro other	-0.21 (0.08)	-0.33 (0.13)	0.235	-0.17 (0.09)	-0.26 (0.13)	0.672	-0.39 (0.11)	-0.47 (0.13)	**0.016**	-24.96 (11.7)	-0.31 (0.14)	0.544
Dem other - Psych	-0.07 (0.08)	-0.11 (0.12)	0.998	-0.06 (0.08)	-0.09 (0.12)	1.000	-0.13 (0.1)	-0.16 (0.12)	0.963	-21.66 (10.79)	-0.26 (0.13)	0.639
Neuro other - Psych	0.14 (0.05)	0.22 (0.08)	0.109	0.11 (0.05)	0.17 (0.08)	0.522	0.25 (0.07)	0.31 (0.08)	**0.005**	3.3 (7.08)	0.04 (0.09)	1.000

Estimates represent post-hoc contrasts from linear regression models with the questionnaire score as outcome variable, diagnosis as predictor of interest, adjusted for age, sex and education. To calculate standardized estimates (presented here as Std. Estimates), the questionnaire scores were z-scored (within each imputed dataset) prior to model fitting. P-values are Tukey-adjusted for multiple comparisons within each model.

SCD = Subjective cognitive decline, MCI = Mild cognitive impairment, AD = Alzheimer's disease dementia, PPA = Primary progressive aphasia, FTD = Frontotemporal neurodegeneration dementia, DLB = Lewy Body disease, CBS/PSP = Corticobasal syndrome / Progressive supranuclear palsy, VaD = Vascular dementia, Dem other = other dementias, Neuro other = other neurological disorders, Psych = Psychiatry

Supplementary Figure 1. Cognition over time

A

MMSE

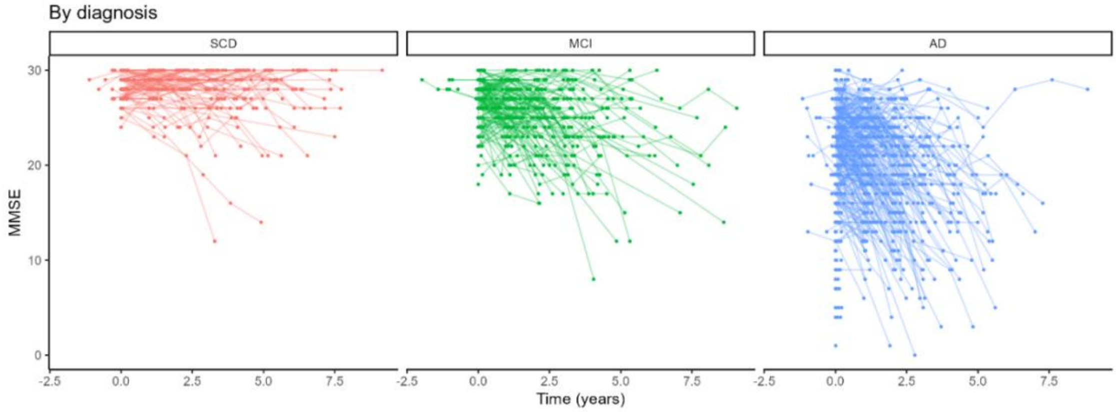

B

Memory

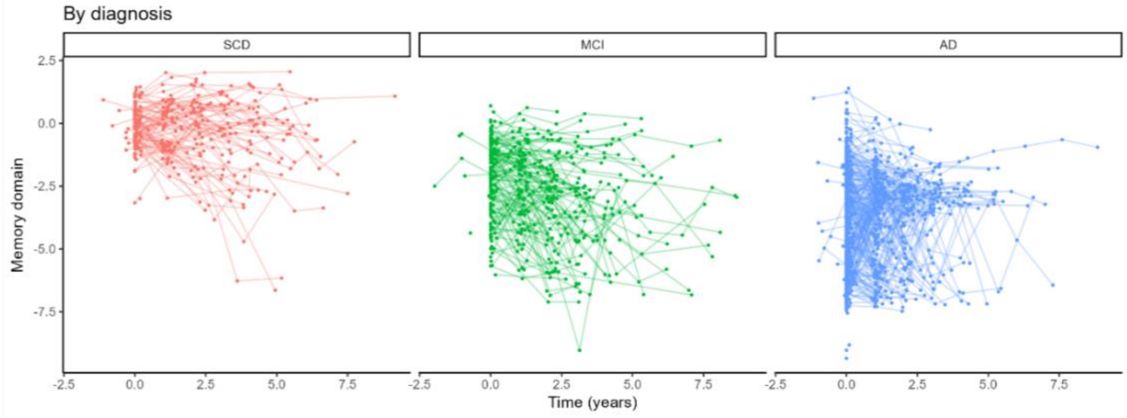

C

Executive function

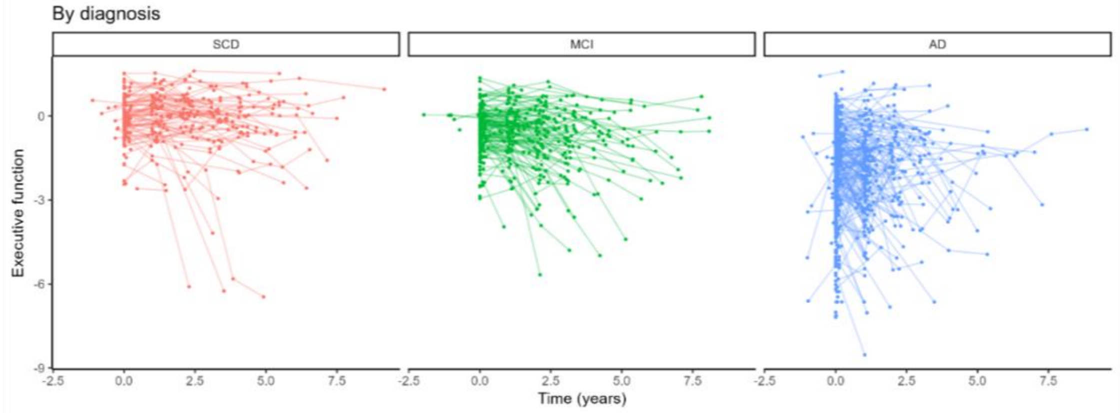

The data depicted in this figure pertain only to participants within the amyloid-positive Alzheimer's Disease (AD) continuum sample. Lines represent individual cognitive trajectories. Sample size: SCD=129, MCI=183, AD=592.

Supplementary Table 6. Longitudinal models tested in part 2 – Cognitive/physical activity and cognitive resilience

Interactive effects models:<br>MMSE / MEM / EF ~ Time * Activity score * Atrophy + Time * Age + Time * Sex (+ Time * Education) + (Time | Subject ID)
Main independent effects models:<br>MMSE / MEM / EF ~ Time * Activity score + Time * Atrophy + Time * Age + Time * Sex (+ Time * Education) + (Time | Subject ID)

Time was measured in years and all other variables were z-scored.
Note that all lower-order terms of an interaction term were also included in all models.
These models were tested only within the amyloid-positive Alzheimer's Disease (AD) continuum sample.

Supplementary Table 7. Linear mixed-effects models – random intercepts and slopes

		MMSE	Memory	Executive function
Lifetime CAQ	All	ri+rs	ri+rs	ri
	SCD	ri+rs	ri+rs	ri+rs
	MCI	ri+rs	ri+rs	ri+rs
	AD	ri+rs	ri	ri
Past CAQ	All	ri+rs	ri+rs	ri
	SCD	ri+rs	ri+rs	ri+rs
	MCI	ri+rs	ri+rs	ri+rs
	AD	ri+rs	ri	ri
Current CAQ	All	ri+rs	ri+rs	ri
	SCD	ri+rs	ri+rs	ri+rs
	MCI	ri+rs	ri+rs	ri+rs
	AD	ri+rs	ri	ri
PASE	All	ri+rs	ri+rs	ri
	SCD	ri+rs	ri+rs	ri+rs
	MCI	ri+rs	ri+rs	ri+rs
	AD	ri+rs	ri	ri

ri = random intercept, ri+rs = random intercepts and random slopes
Random intercept only models were fitted when random slopes were not possible to model due to the number of observations being smaller than the number of total random effects.
These models were tested only within the amyloid-positive Alzheimer's Disease (AD) continuum sample.

Supplementary Table 8a. Cross-sectional main effects on cognition independent of temporoparietal cortical thickness, adjusted for education, operationalized as tertiles

			MMSE			Memory			Executive function		
			Estimate [CI]	p	pFDR	Estimate [CI]	p	pFDR	Estimate [CI]	p	pFDR
β Questionnaire score	Lifetime CAQ	All	0.123 [0.064 - 0.181]	**<0.001**	***	0.038 [-0.032 - 0.107]	0.29		0.138 [0.073 - 0.203]	**<0.001**	***
		SCD	0.005 [-0.041 - 0.051]	0.832		0.033 [-0.035 - 0.1]	0.344		0.152 [0.067 - 0.237]	**0.001**	**
		MCI	-0.003 [-0.075 - 0.069]	0.943		-0.084 [-0.199 - 0.031]	0.151		0.064 [-0.012 - 0.139]	0.097	
		AD	0.143 [0.065 - 0.221]	**<0.001**	**	0.001 [-0.079 - 0.08]	0.984		0.118 [0.029 - 0.207]	**0.01**	
	Past CAQ	All	0.072 [0.014 - 0.129]	**0.015**		0.008 [-0.061 - 0.077]	0.815		0.091 [0.027 - 0.156]	**0.006**	
		SCD	0.003 [-0.042 - 0.048]	0.887		0.032 [-0.034 - 0.098]	0.339		0.138 [0.054 - 0.221]	**0.001**	*
		MCI	-0.007 [-0.078 - 0.063]	0.834		-0.087 [-0.199 - 0.025]	0.128		0.055 [-0.019 - 0.128]	0.145	
		AD	0.087 [0.009 - 0.165]	**0.029**		-0.015 [-0.095 - 0.064]	0.704		0.071 [-0.018 - 0.159]	0.118	
	Current CAQ	All	0.218 [0.163 - 0.272]	**<0.001**	***	0.108 [0.041 - 0.175]	**0.002**	**	0.219 [0.158 - 0.281]	**<0.001**	***
		SCD	0.011 [-0.043 - 0.065]	0.681		0.024 [-0.057 - 0.104]	0.564		0.164 [0.062 - 0.265]	**0.002**	*
		MCI	0.008 [-0.065 - 0.081]	0.828		-0.037 [-0.156 - 0.081]	0.537		0.064 [-0.012 - 0.14]	0.097	
		AD	0.23 [0.158 - 0.302]	**<0.001**	***	0.045 [-0.03 - 0.12]	0.24		0.198 [0.116 - 0.281]	**<0.001**	***
	PASE	All	0.078 [0.028 - 0.129]	**0.002**	*	0.025 [-0.034 - 0.085]	0.404		0.084 [0.029 - 0.14]	**0.003**	*
		SCD	-0.013 [-0.053 - 0.028]	0.533		0.023 [-0.037 - 0.083]	0.458		0.051 [-0.028 - 0.129]	0.206	
		MCI	-0.022 [-0.079 - 0.034]	0.438		-0.054 [-0.146 - 0.038]	0.249		-0.018 [-0.078 - 0.043]	0.565	
		AD	0.105 [0.036 - 0.175]	**0.003**	*	0.02 [-0.05 - 0.09]	0.574		0.102 [0.025 - 0.179]	**0.009**	

Coefficients from linear mixed-effect models, adjusted for age and sex and **education (tertiles).**
Analyses presented in this table were conducted exclusively within the amyloid-positive Alzheimer's Disease (AD) continuum sample.
Stars represent FDR adjusted p-values: * = $p_{FDR} < 0.05$, ** = $p_{FDR} < 0.01$, *** = $p_{FDR} < 0.001$

Supplementary Table 8b. Cross-sectional main effects on cognition independent of temporoparietal cortical thickness, adjusted for education, operationalized as (Verhage-transformed) years of education.

			MMSE			Memory			Executive function		
			Estimate [CI]	p	pFDR	Estimate [CI]	p	pFDR	Estimate [CI]	p	pFDR
β Questionnaire score	Lifetime CAQ	All	0.117 [0.06 - 0.175]	**<0.001**	***	0.033 [-0.036 - 0.102]	0.344		0.136 [0.072 - 0.2]	**<0.001**	***
		SCD	0.011 [-0.036 - 0.057]	0.659		0.038 [-0.03 - 0.106]	0.275		0.155 [0.07 - 0.24]	**<0.001**	**
		MCI	-0.013 [-0.083 - 0.058]	0.725		-0.083 [-0.197 - 0.03]	0.15		0.081 [0.005 - 0.157]	**0.037**	
		AD	0.135 [0.057 - 0.212]	**0.001**	**	-0.009 [-0.088 - 0.069]	0.817		0.11 [0.022 - 0.197]	**0.014**	
	Past CAQ	All	0.069 [0.012 - 0.126]	**0.018**		0.007 [-0.061 - 0.074]	0.849		0.092 [0.029 - 0.156]	**0.004**	
		SCD	0.009 [-0.037 - 0.055]	0.694		0.037 [-0.029 - 0.103]	0.27		0.141 [0.058 - 0.225]	**0.001**	*
		MCI	-0.018 [-0.086 - 0.051]	0.611		-0.087 [-0.197 - 0.024]	0.125		0.071 [-0.004 - 0.145]	0.062	
		AD	0.082 [0.005 - 0.159]	**0.037**		-0.023 [-0.101 - 0.055]	0.563		0.066 [-0.021 - 0.153]	0.134	
	Current CAQ	All	0.211 [0.156 - 0.266]	**<0.001**	***	0.1 [0.033 - 0.168]	**0.003**	*	0.213 [0.151 - 0.276]	**<0.001**	***
		SCD	0.013 [-0.042 - 0.069]	0.637		0.027 [-0.054 - 0.108]	0.511		0.163 [0.061 - 0.265]	**0.002**	*
		MCI	0.004 [-0.067 - 0.076]	0.908		-0.034 [-0.152 - 0.083]	0.565		0.076 [0 - 0.153]	0.05	
		AD	0.222 [0.15 - 0.295]	**<0.001**	***	0.034 [-0.041 - 0.109]	0.377		0.187 [0.104 - 0.27]	**<0.001**	***
	PASE	All	0.09 [0.04 - 0.14]	**<0.001**	**	0.033 [-0.027 - 0.093]	0.276		0.097 [0.042 - 0.153]	**0.001**	**
		SCD	-0.005 [-0.046 - 0.036]	0.804		0.029 [-0.03 - 0.089]	0.336		0.063 [-0.014 - 0.14]	0.11	
		MCI	-0.013 [-0.07 - 0.043]	0.644		-0.047 [-0.139 - 0.044]	0.311		0.002 [-0.059 - 0.064]	0.939	
		AD	0.115 [0.046 - 0.184]	**0.001**	*	0.026 [-0.043 - 0.095]	0.463		0.114 [0.037 - 0.191]	**0.004**	*

Coefficients from linear mixed-effect models, adjusted for age and sex and **education** (**years**).

Analyses presented in this table were conducted exclusively within the amyloid-positive Alzheimer's Disease (AD) continuum sample.

Stars represent FDR adjusted p-values: * = $p_{FDR} < 0.05$, ** = $p_{FDR} < 0.01$, *** = $p_{FDR} < 0.001$

Supplementary Table 8c. Cross-sectional main effects on cognition independent of temporoparietal cortical thickness, adjusted for education, operationalized as tertiles, and for APOE-ε4 carriership status

			MMSE			Memory			Executive function		
			Estimate [CI]	p	pFDR	Estimate [CI]	p	pFDR	Estimate [CI]	p	pFDR
β Questionnaire score	Lifetime CAQ	All	0.123 [0.065 - 0.181]	**<0.001**	***	0.04 [-0.029 - 0.109]	0.26		0.138 [0.073 - 0.202]	**<0.001**	***
		SCD	0.006 [-0.041 - 0.053]	0.794		0.029 [-0.038 - 0.096]	0.4		0.159 [0.074 - 0.243]	**<0.001**	**
		MCI	-0.002 [-0.073 - 0.07]	0.96		-0.083 [-0.198 - 0.031]	0.154		0.064 [-0.011 - 0.14]	0.095	
		AD	0.144 [0.066 - 0.223]	**<0.001**	**	0.006 [-0.074 - 0.085]	0.89		0.114 [0.025 - 0.203]	**0.012**	
	Past CAQ	All	0.071 [0.014 - 0.129]	**0.015**		0.009 [-0.059 - 0.078]	0.793		0.092 [0.028 - 0.156]	**0.005**	
		SCD	0.004 [-0.041 - 0.05]	0.848		0.028 [-0.037 - 0.094]	0.397		0.145 [0.061 - 0.228]	**0.001**	**
		MCI	-0.008 [-0.078 - 0.061]	0.811		-0.088 [-0.2 - 0.024]	0.122		0.056 [-0.018 - 0.129]	0.139	
		AD	0.088 [0.01 - 0.166]	**0.027**		-0.012 [-0.091 - 0.067]	0.775		0.068 [-0.021 - 0.156]	0.135	
	Current CAQ	All	0.219 [0.165 - 0.274]	**<0.001**	***	0.114 [0.047 - 0.181]	0.001	**	0.217 [0.155 - 0.279]	**<0.001**	***
		SCD	0.012 [-0.042 - 0.067]	0.663		0.021 [-0.058 - 0.1]	0.6		0.167 [0.067 - 0.268]	**0.001**	*
		MCI	0.015 [-0.057 - 0.088]	0.679		-0.03 [-0.149 - 0.089]	0.619		0.064 [-0.013 - 0.14]	0.102	
		AD	0.232 [0.16 - 0.304]	**<0.001**	***	0.051 [-0.024 - 0.126]	0.185		0.194 [0.111 - 0.277]	**<0.001**	***
	PASE	All	0.08 [0.03 - 0.131]	**0.002**	*	0.031 [-0.028 - 0.091]	0.305		0.081 [0.025 - 0.137]	**0.004**	*
		SCD	-0.013 [-0.054 - 0.028]	0.544		0.031 [-0.029 - 0.09]	0.312		0.046 [-0.032 - 0.125]	0.25	
		MCI	-0.022 [-0.079 - 0.034]	0.441		-0.056 [-0.148 - 0.036]	0.234		-0.018 [-0.079 - 0.042]	0.552	
		AD	0.108 [0.039 - 0.178]	**0.002**	*	0.026 [-0.043 - 0.096]	0.46		0.097 [0.019 - 0.174]	**0.014**	

Coefficients from linear mixed-effect models, adjusted for age and sex, **education (tertiles) and APOE- ε4 carriership.**
Analyses presented in this table were conducted exclusively within the amyloid-positive Alzheimer's Disease (AD) continuum sample.
Stars represent FDR adjusted p-values: * = $p_{FDR} < 0.05$, ** = $p_{FDR} < 0.01$, *** = $p_{FDR} < 0.001$

Supplementary Table 9a. Longitudinal main effects on cognition independent of temporoparietal cortical thickness

			MMSE			Memory			Executive function		
			Estimate [CI]	p	pFDR	Estimate [CI]	p	pFDR	Estimate [CI]	p	pFDR
β Time × Questionnaire score	Lifetime CAQ	All	0.005 [-0.026 - 0.035]	0.771		0.001 [-0.031 - 0.032]	0.96		-0.011 [-0.034 - 0.013]	0.381	
		SCD	-0.007 [-0.043 - 0.029]	0.705		-0.009 [-0.056 - 0.038]	0.696		-0.039 [-0.109 - 0.031]	0.279	
		MCI	-0.005 [-0.05 - 0.04]	0.83		0.01 [-0.036 - 0.056]	0.672		0.003 [-0.044 - 0.05]	0.903	
		AD	-0.007 [-0.059 - 0.045]	0.792		0.029 [-0.016 - 0.074]	0.211		0.04 [-0.021 - 0.1]	0.201	
	Past CAQ	All	-0.001 [-0.031 - 0.029]	0.939		0.006 [-0.025 - 0.037]	0.709		-0.007 [-0.03 - 0.016]	0.539	
		SCD	-0.007 [-0.042 - 0.028]	0.685		-0.01 [-0.056 - 0.036]	0.662		-0.039 [-0.107 - 0.029]	0.264	
		MCI	-0.013 [-0.057 - 0.031]	0.57		0.008 [-0.036 - 0.053]	0.714		0.007 [-0.039 - 0.053]	0.752	
		AD	-0.004 [-0.055 - 0.047]	0.871		0.033 [-0.013 - 0.079]	0.163		0.04 [-0.021 - 0.1]	0.202	
	Current CAQ	All	0.021 [-0.01 - 0.051]	0.181		-0.016 [-0.048 - 0.016]	0.319		-0.016 [-0.041 - 0.009]	0.212	
		SCD	-0.002 [-0.045 - 0.04]	0.915		-0.002 [-0.057 - 0.054]	0.945		-0.022 [-0.108 - 0.063]	0.607	
		MCI	0.026 [-0.02 - 0.073]	0.264		0.01 [-0.038 - 0.058]	0.683		-0.014 [-0.061 - 0.034]	0.578	
		AD	-0.013 [-0.062 - 0.037]	0.612		0.007 [-0.036 - 0.049]	0.758		0.03 [-0.031 - 0.09]	0.34	
	PASE	All	0.009 [-0.017 - 0.034]	0.501		-0.012 [-0.039 - 0.015]	0.374		0.009 [-0.01 - 0.029]	0.359	
		SCD	0.006 [-0.029 - 0.041]	0.737		0.008 [-0.037 - 0.053]	0.729		0.023 [-0.046 - 0.092]	0.516	
		MCI	0.002 [-0.033 - 0.037]	0.922		0.001 [-0.035 - 0.038]	0.948		0.025 [-0.011 - 0.061]	0.181	
		AD	-0.004 [-0.048 - 0.04]	0.855		0.01 [-0.026 - 0.046]	0.571		-0.003 [-0.047 - 0.041]	0.891	

Coefficients from linear mixed-effect models, adjusted for age and sex.

Analyses presented in this table were conducted exclusively within the amyloid-positive Alzheimer's Disease (AD) continuum sample.

Stars represent FDR adjusted p-values: * = $p_{FDR} < 0.05$, ** = $p_{FDR} < 0.01$, *** = $p_{FDR} < 0.001$

Supplementary Table 9b. Longitudinal main effects on cognition independent of temporoparietal cortical thickness, adjusted for education, operationalized as tertiles

			MMSE			Memory			Executive function		
			Estimate [CI]	p	pFDR	Estimate [CI]	p	pFDR	Estimate [CI]	p	pFDR
β Time × Questionnaire score	Lifetime CAQ	All	0.005 [-0.026 - 0.035]	0.771		0.001 [-0.031 - 0.032]	0.96		-0.011 [-0.034 - 0.013]	0.381	
		SCD	-0.007 [-0.043 - 0.029]	0.705		-0.009 [-0.056 - 0.038]	0.696		-0.039 [-0.109 - 0.031]	0.279	
		MCI	-0.005 [-0.05 - 0.04]	0.83		0.01 [-0.036 - 0.056]	0.672		0.003 [-0.044 - 0.05]	0.903	
		AD	-0.007 [-0.059 - 0.045]	0.792		0.029 [-0.016 - 0.074]	0.211		0.04 [-0.021 - 0.1]	0.201	
	Past CAQ	All	-0.001 [-0.031 - 0.029]	0.939		0.006 [-0.025 - 0.037]	0.709		-0.007 [-0.03 - 0.016]	0.539	
		SCD	-0.007 [-0.042 - 0.028]	0.685		-0.01 [-0.056 - 0.036]	0.662		-0.039 [-0.107 - 0.029]	0.264	
		MCI	-0.013 [-0.057 - 0.031]	0.57		0.008 [-0.036 - 0.053]	0.714		0.007 [-0.039 - 0.053]	0.752	
		AD	-0.004 [-0.055 - 0.047]	0.871		0.033 [-0.013 - 0.079]	0.163		0.04 [-0.021 - 0.1]	0.202	
	Current CAQ	All	0.021 [-0.01 - 0.051]	0.181		-0.016 [-0.048 - 0.016]	0.319		-0.016 [-0.041 - 0.009]	0.212	
		SCD	-0.002 [-0.045 - 0.04]	0.915		-0.002 [-0.057 - 0.054]	0.945		-0.022 [-0.108 - 0.063]	0.607	
		MCI	0.026 [-0.02 - 0.073]	0.264		0.01 [-0.038 - 0.058]	0.683		-0.014 [-0.061 - 0.034]	0.578	
		AD	-0.013 [-0.062 - 0.037]	0.612		0.007 [-0.036 - 0.049]	0.758		0.03 [-0.031 - 0.09]	0.34	
	PASE	All	0.009 [-0.017 - 0.034]	0.501		-0.012 [-0.039 - 0.015]	0.374		0.009 [-0.01 - 0.029]	0.359	
		SCD	0.006 [-0.029 - 0.041]	0.737		0.008 [-0.037 - 0.053]	0.729		0.023 [-0.046 - 0.092]	0.516	
		MCI	0.002 [-0.033 - 0.037]	0.922		0.001 [-0.035 - 0.038]	0.948		0.025 [-0.011 - 0.061]	0.181	
		AD	-0.004 [-0.048 - 0.04]	0.855		0.01 [-0.026 - 0.046]	0.571		-0.003 [-0.047 - 0.041]	0.891	

Coefficients from linear mixed-effect models, adjusted for age, sex and **education (tertiles)**
Analyses presented in this table were conducted exclusively within the amyloid-positive Alzheimer's Disease (AD) continuum sample.
Stars represent FDR adjusted p-values: * = $p_{FDR} < 0.05$, ** = $p_{FDR} < 0.01$, *** = $p_{FDR} < 0.001$

Supplementary Table 9c. Longitudinal main effects on cognition independent of temporoparietal cortical thickness, adjusted for education, operationalized as (Verhage-transformed) years of education.

			MMSE			Memory			Executive function		
			Estimate [CI]	p	pFDR	Estimate [CI]	p	pFDR	Estimate [CI]	p	pFDR
β Time × Questionnaire score	Lifetime CAQ	All	0.002 [-0.028 - 0.031]	0.909		0.004 [-0.027 - 0.034]	0.82		-0.008 [-0.031 - 0.014]	0.459	
		SCD	-0.004 [-0.041 - 0.033]	0.834		-0.009 [-0.056 - 0.037]	0.701		-0.031 [-0.102 - 0.04]	0.394	
		MCI	0.004 [-0.04 - 0.048]	0.856		0.012 [-0.031 - 0.055]	0.588		0.008 [-0.038 - 0.054]	0.722	
		AD	-0.019 [-0.07 - 0.032]	0.469		0.042 [0 - 0.085]	0.053		0.029 [-0.03 - 0.088]	0.33	
	Past CAQ	All	-0.003 [-0.032 - 0.026]	0.828		0.008 [-0.023 - 0.038]	0.621		-0.005 [-0.027 - 0.016]	0.621	
		SCD	-0.006 [-0.042 - 0.03]	0.757		-0.011 [-0.056 - 0.035]	0.64		-0.033 [-0.101 - 0.036]	0.351	
		MCI	-0.004 [-0.047 - 0.039]	0.855		0.01 [-0.032 - 0.053]	0.627		0.012 [-0.033 - 0.057]	0.602	
		AD	-0.015 [-0.065 - 0.035]	0.556		0.044 [0.001 - 0.087]	0.045		0.028 [-0.031 - 0.087]	0.351	
	Current CAQ	All	0.017 [-0.013 - 0.047]	0.257		-0.012 [-0.043 - 0.02]	0.466		-0.015 [-0.04 - 0.01]	0.247	
		SCD	0.006 [-0.038 - 0.051]	0.774		0.002 [-0.053 - 0.057]	0.934		-0.008 [-0.095 - 0.078]	0.851	
		MCI	0.032 [-0.013 - 0.078]	0.165		0.011 [-0.036 - 0.059]	0.634		-0.008 [-0.055 - 0.039]	0.738	
		AD	-0.022 [-0.071 - 0.027]	0.389		0.021 [-0.021 - 0.062]	0.329		0.028 [-0.033 - 0.089]	0.369	
	PASE	All	0.01 [-0.016 - 0.035]	0.46		-0.012 [-0.038 - 0.015]	0.379		0.01 [-0.009 - 0.029]	0.311	
		SCD	0.011 [-0.026 - 0.047]	0.561		0.013 [-0.032 - 0.058]	0.577		0.03 [-0.041 - 0.101]	0.409	
		MCI	0.006 [-0.03 - 0.041]	0.752		0.001 [-0.035 - 0.037]	0.94		0.029 [-0.007 - 0.065]	0.115	
		AD	-0.007 [-0.05 - 0.037]	0.767		0.009 [-0.026 - 0.044]	0.603		-0.011 [-0.053 - 0.032]	0.616	

Coefficients from linear mixed-effect models, adjusted for age, sex and **education (years)**
Analyses presented in this table were conducted exclusively within the amyloid-positive Alzheimer's Disease (AD) continuum sample.
Stars represent FDR adjusted p-values: * = $p_{FDR} < 0.05$, ** = $p_{FDR} < 0.01$, *** = $p_{FDR} < 0.001$

Supplementary Table 9d. Longitudinal main effects on cognition independent of temporoparietal cortical thickness, adjusted for education, operationalized as tertiles, and for APOE-ε4 carriership status

			MMSE			Memory			Executive function		
			Estimate [CI]	p	pFDR	Estimate [CI]	p	pFDR	Estimate [CI]	p	pFDR
β Time × Questionnaire score	Lifetime CAQ	All	0.004 [-0.027 - 0.034]	0.808		-0.001 [-0.032 - 0.031]	0.965		-0.012 [-0.035 - 0.012]	0.33	
		SCD	-0.01 [-0.046 - 0.027]	0.605		-0.016 [-0.064 - 0.032]	0.503		-0.035 [-0.107 - 0.037]	0.336	
		MCI	-0.005 [-0.05 - 0.041]	0.844		0.011 [-0.035 - 0.057]	0.641		0.004 [-0.043 - 0.051]	0.871	
		AD	-0.008 [-0.06 - 0.044]	0.769		0.029 [-0.016 - 0.075]	0.208		0.041 [-0.02 - 0.101]	0.189	
	Past CAQ	All	-0.002 [-0.032 - 0.028]	0.897		0.005 [-0.027 - 0.036]	0.776		-0.009 [-0.032 - 0.014]	0.457	
		SCD	-0.01 [-0.046 - 0.026]	0.589		-0.017 [-0.064 - 0.03]	0.476		-0.036 [-0.106 - 0.034]	0.316	
		MCI	-0.012 [-0.057 - 0.032]	0.587		0.009 [-0.036 - 0.054]	0.697		0.007 [-0.038 - 0.053]	0.751	
		AD	-0.005 [-0.056 - 0.046]	0.846		0.033 [-0.013 - 0.079]	0.161		0.041 [-0.02 - 0.101]	0.19	
	Current CAQ	All	0.02 [-0.01 - 0.051]	0.184		-0.017 [-0.049 - 0.014]	0.289		-0.015 [-0.04 - 0.01]	0.235	
		SCD	-0.005 [-0.048 - 0.039]	0.837		-0.007 [-0.063 - 0.048]	0.799		-0.018 [-0.105 - 0.069]	0.681	
		MCI	0.026 [-0.021 - 0.073]	0.272		0.013 [-0.036 - 0.062]	0.602		-0.01 [-0.058 - 0.038]	0.691	
		AD	-0.013 [-0.062 - 0.037]	0.61		0.007 [-0.036 - 0.049]	0.757		0.03 [-0.03 - 0.091]	0.328	
	PASE	All	0.009 [-0.017 - 0.035]	0.49		-0.012 [-0.039 - 0.014]	0.369		0.009 [-0.01 - 0.029]	0.351	
		SCD	0.008 [-0.028 - 0.043]	0.67		0.015 [-0.031 - 0.061]	0.515		0.019 [-0.052 - 0.09]	0.607	
		MCI	0.001 [-0.034 - 0.037]	0.934		0.002 [-0.034 - 0.039]	0.908		0.025 [-0.01 - 0.061]	0.166	
		AD	-0.005 [-0.048 - 0.039]	0.838		0.01 [-0.026 - 0.046]	0.579		-0.004 [-0.048 - 0.04]	0.872	

Coefficients from linear mixed-effect models, adjusted for age, sex, **education (tertiles) and APOE- ε4 carriership.**
Analyses presented in this table were conducted exclusively within the amyloid-positive Alzheimer's Disease (AD) continuum sample.
Stars represent FDR adjusted p-values: * = $p_{FDR} < 0.05$, ** = $p_{FDR} < 0.01$, *** = $p_{FDR} < 0.001$

Supplementary Table 10a. Two-way interaction effect of cognitive/physical activity with temporoparietal cortical thickness on baseline cognition

			MMSE			Memory			Executive function		
			Estimate [CI]	p	pFDR	Estimate [CI]	p	pFDR	Estimate [CI]	p	pFDR
β TP thickness × Questionnaire score	Lifetime CAQ	All	-0.022 [-0.075 - 0.03]	0.403		0.027 [-0.037 - 0.091]	0.408		-0.012 [-0.071 - 0.048]	0.699	
		SCD	-0.007 [-0.078 - 0.063]	0.841		-0.042 [-0.138 - 0.053]	0.384		-0.033 [-0.16 - 0.094]	0.611	
		MCI	0.084 [-0.01 - 0.178]	0.081		0.143 [-0.006 - 0.292]	0.059		-0.002 [-0.108 - 0.104]	0.976	
		AD	-0.004 [-0.08 - 0.073]	0.922		-0.023 [-0.103 - 0.056]	0.566		-0.024 [-0.112 - 0.064]	0.593	
	Past CAQ	All	0.006 [-0.046 - 0.058]	0.83		0.019 [-0.044 - 0.082]	0.553		0.014 [-0.046 - 0.073]	0.654	
		SCD	-0.01 [-0.079 - 0.06]	0.788		-0.059 [-0.153 - 0.035]	0.218		-0.038 [-0.166 - 0.089]	0.554	
		MCI	0.086 [-0.006 - 0.177]	0.067		0.133 [-0.012 - 0.278]	0.071		-0.004 [-0.108 - 0.1]	0.938	
		AD	0.026 [-0.05 - 0.101]	0.506		-0.025 [-0.104 - 0.054]	0.532		0.01 [-0.077 - 0.098]	0.819	
	Current CAQ	All	-0.071 [-0.12 - -0.021]	**0.005**	*	0.058 [-0.008 - 0.124]	0.085		-0.07 [-0.129 - -0.011]	**0.02**	*
		SCD	0.012 [-0.061 - 0.085]	0.745		0.043 [-0.056 - 0.142]	0.397		0.023 [-0.105 - 0.152]	0.72	
		MCI	0.035 [-0.067 - 0.137]	0.497		0.104 [-0.055 - 0.264]	0.2		0.012 [-0.102 - 0.127]	0.833	
		AD	-0.065 [-0.135 - 0.006]	0.071		0.001 [-0.078 - 0.081]	0.972		-0.11 [-0.194 - -0.026]	**0.01**	*
	PASE	All	-0.067 [-0.113 - -0.021]	**0.004**	*	-0.01 [-0.065 - 0.044]	0.707		-0.056 [-0.107 - -0.004]	**0.035**	
		SCD	-0.027 [-0.073 - 0.019]	0.252		-0.002 [-0.065 - 0.061]	0.943		-0.066 [-0.151 - 0.019]	0.13	
		MCI	0.041 [-0.045 - 0.128]	0.348		-0.035 [-0.173 - 0.102]	0.615		-0.071 [-0.173 - 0.032]	0.176	
		AD	-0.071 [-0.148 - 0.006]	0.069		-0.01 [-0.089 - 0.068]	0.794		-0.041 [-0.127 - 0.045]	0.352	

Coefficients from linear mixed-effect models, adjusted for age and sex

Analyses presented in this table were conducted exclusively within the amyloid-positive Alzheimer's Disease (AD) continuum sample.

Stars represent FDR adjusted p-values: * = $p_{FDR} < 0.05$, ** = $p_{FDR} < 0.01$, *** = $p_{FDR} < 0.001$

Supplementary Table 10b. Two-way interaction effect of cognitive/physical activity with temporoparietal cortical thickness on baseline cognition, adjusted for education, operationalized as tertiles

			MMSE			Memory			Executive function		
			Estimate [CI]	p	pFDR	Estimate [CI]	p	pFDR	Estimate [CI]	p	pFDR
β Time × TP thickness × Questionnaire score	Lifetime CAQ	All	-0.019 [-0.071 - 0.033]	0.483		0.029 [-0.035 - 0.093]	0.375		-0.009 [-0.068 - 0.05]	0.763	
		SCD	-0.014 [-0.079 - 0.051]	0.666		-0.049 [-0.143 - 0.045]	0.308		-0.044 [-0.164 - 0.076]	0.476	
		MCI	0.089 [-0.005 - 0.183]	0.063		0.151 [0.001 - 0.3]	**0.048**		0.007 [-0.094 - 0.107]	0.897	
		AD	-0.001 [-0.077 - 0.074]	0.972		-0.024 [-0.104 - 0.055]	0.546		-0.029 [-0.116 - 0.059]	0.519	
	Past CAQ	All	0.012 [-0.04 - 0.063]	0.652		0.022 [-0.041 - 0.085]	0.493		0.019 [-0.039 - 0.078]	0.515	
		SCD	-0.017 [-0.081 - 0.046]	0.592		-0.067 [-0.159 - 0.025]	0.153		-0.053 [-0.172 - 0.067]	0.389	
		MCI	0.091 [-0.001 - 0.182]	0.051		0.141 [-0.005 - 0.286]	0.058		0.005 [-0.093 - 0.103]	0.921	
		AD	0.03 [-0.045 - 0.104]	0.434		-0.028 [-0.106 - 0.051]	0.488		0.007 [-0.08 - 0.094]	0.879	
	Current CAQ	All	-0.077 [-0.126 - -0.028]	**0.002**	**	0.055 [-0.01 - 0.121]	0.099		-0.078 [-0.135 - -0.02]	**0.008**	*
		SCD	0.007 [-0.06 - 0.075]	0.83		0.042 [-0.055 - 0.14]	0.393		0.021 [-0.102 - 0.143]	0.74	
		MCI	0.038 [-0.064 - 0.139]	0.466		0.106 [-0.054 - 0.266]	0.194		0.014 [-0.092 - 0.119]	0.8	
		AD	-0.072 [-0.142 - -0.002]	**0.043**		0.004 [-0.076 - 0.084]	0.923		-0.116 [-0.199 - -0.032]	**0.007**	*
	PASE	All	-0.064 [-0.109 - -0.02]	**0.004**	*	-0.003 [-0.057 - 0.051]	0.905		-0.05 [-0.1 - 0]	**0.049**	
		SCD	-0.024 [-0.066 - 0.018]	0.264		0 [-0.061 - 0.061]	0.995		-0.061 [-0.14 - 0.018]	0.129	
		MCI	0.035 [-0.053 - 0.123]	0.437		-0.052 [-0.193 - 0.09]	0.474		-0.078 [-0.171 - 0.015]	0.1	
		AD	-0.072 [-0.146 - 0.002]	0.057		-0.014 [-0.091 - 0.064]	0.732		-0.043 [-0.128 - 0.041]	0.312	

Coefficients from linear mixed-effect models, adjusted for age, sex and **education (tertiles).**
Analyses presented in this table were conducted exclusively within the amyloid-positive Alzheimer's Disease (AD) continuum sample.
Stars represent FDR adjusted p-values: * = $p_{FDR} < 0.05$, ** = $p_{FDR} < 0.01$, *** = $p_{FDR} < 0.001$

Supplementary Table 10c. Two-way interaction effect of cognitive/physical activity with temporoparietal cortical thickness on baseline cognition, adjusted for education, operationalized as (Verhage-transformed) years of education.

			MMSE			Memory			Executive function		
			Estimate [CI]	p	pFDR	Estimate [CI]	p	pFDR	Estimate [CI]	p	pFDR
β Time × TP thickness × Questionnaire score	Lifetime CAQ	All	-0.026 [-0.077 - 0.026]	0.326		0.025 [-0.038 - 0.088]	0.438		-0.016 [-0.074 - 0.043]	0.601	
		SCD	-0.029 [-0.095 - 0.038]	0.399		-0.061 [-0.156 - 0.034]	0.21		-0.066 [-0.187 - 0.054]	0.279	
		MCI	0.085 [-0.007 - 0.178]	0.071		0.143 [-0.005 - 0.291]	0.059		0 [-0.101 - 0.101]	0.995	
		AD	-0.01 [-0.085 - 0.065]	0.798		-0.026 [-0.105 - 0.053]	0.513		-0.033 [-0.12 - 0.054]	0.459	
	Past CAQ	All	0.003 [-0.048 - 0.054]	0.91		0.018 [-0.044 - 0.081]	0.57		0.011 [-0.047 - 0.069]	0.7	
		SCD	-0.031 [-0.096 - 0.035]	0.359		-0.078 [-0.171 - 0.015]	0.101		-0.074 [-0.194 - 0.047]	0.228	
		MCI	0.087 [-0.004 - 0.177]	0.06		0.133 [-0.012 - 0.277]	0.071		-0.003 [-0.102 - 0.096]	0.957	
		AD	0.019 [-0.055 - 0.093]	0.612		-0.029 [-0.107 - 0.049]	0.46		0.001 [-0.085 - 0.088]	0.973	
	Current CAQ	All	-0.081 [-0.13 - -0.032]	**0.001**	**	0.051 [-0.015 - 0.117]	0.127		-0.082 [-0.139 - -0.024]	**0.005**	*
		SCD	-0.004 [-0.073 - 0.064]	0.902		0.031 [-0.067 - 0.129]	0.536		0.001 [-0.121 - 0.124]	0.985	
		MCI	0.037 [-0.064 - 0.137]	0.475		0.105 [-0.055 - 0.264]	0.199		0.015 [-0.092 - 0.122]	0.785	
		AD	-0.074 [-0.144 - -0.004]	**0.037**		0.001 [-0.079 - 0.081]	0.98		-0.118 [-0.201 - -0.035]	**0.005**	*
	PASE	All	-0.063 [-0.107 - -0.019]	**0.005**	*	-0.006 [-0.059 - 0.048]	0.833		-0.05 [-0.1 - -0.001]	**0.046**	
		SCD	-0.028 [-0.071 - 0.016]	0.209		-0.003 [-0.065 - 0.058]	0.911		-0.068 [-0.146 - 0.011]	0.091	
		MCI	0.047 [-0.038 - 0.133]	0.275		-0.033 [-0.171 - 0.105]	0.634		-0.051 [-0.145 - 0.043]	0.286	
		AD	-0.074 [-0.148 - 0]	**0.049**		-0.013 [-0.09 - 0.065]	0.748		-0.046 [-0.13 - 0.037]	0.279	

Coefficients from linear mixed-effect models, adjusted for age, sex **and education (years).**
Analyses presented in this table were conducted exclusively within the amyloid-positive Alzheimer's Disease (AD) continuum sample.
Stars represent FDR adjusted p-values: * = $p_{FDR} < 0.05$, ** = $p_{FDR} < 0.01$, *** = $p_{FDR} < 0.001$

Supplementary Table 10d. Two-way interaction effect of cognitive/physical activity with temporoparietal cortical thickness on baseline cognition, adjusted for education, operationalized as tertiles, and for APOE-ε4 carriership status

			MMSE			Memory			Executive function		
			Estimate [CI]	p	pFDR	Estimate [CI]	p	pFDR	Estimate [CI]	p	pFDR
β Time × TP thickness × Questionnaire score	Lifetime CAQ	All	-0.019 [-0.071 - 0.033]	0.477		0.026 [-0.038 - 0.089]	0.427		-0.008 [-0.066 - 0.051]	0.79	
		SCD	-0.014 [-0.08 - 0.051]	0.665		-0.045 [-0.138 - 0.049]	0.347		-0.047 [-0.166 - 0.072]	0.438	
		MCI	0.089 [-0.005 - 0.182]	0.063		0.153 [0.003 - 0.302]	0.045		0.007 [-0.093 - 0.107]	0.893	
		AD	-0.001 [-0.077 - 0.075]	0.979		-0.026 [-0.105 - 0.053]	0.522		-0.029 [-0.116 - 0.058]	0.513	
	Past CAQ	All	0.012 [-0.04 - 0.063]	0.655		0.019 [-0.043 - 0.082]	0.545		0.02 [-0.038 - 0.079]	0.494	
		SCD	-0.017 [-0.081 - 0.046]	0.593		-0.066 [-0.158 - 0.025]	0.154		-0.052 [-0.171 - 0.067]	0.39	
		MCI	0.09 [-0.001 - 0.181]	0.052		0.142 [-0.004 - 0.287]	0.056		0.005 [-0.093 - 0.103]	0.918	
		AD	0.03 [-0.044 - 0.105]	0.426		-0.028 [-0.106 - 0.05]	0.484		0.006 [-0.082 - 0.093]	0.901	
	Current CAQ	All	-0.078 [-0.126 - -0.029]	**0.002**	**	0.052 [-0.013 - 0.117]	0.117		-0.077 [-0.134 - -0.02]	**0.009**	*
		SCD	0.007 [-0.062 - 0.076]	0.84		0.057 [-0.04 - 0.154]	0.247		0.007 [-0.116 - 0.13]	0.91	
		MCI	0.039 [-0.062 - 0.14]	0.449		0.11 [-0.049 - 0.27]	0.176		0.014 [-0.091 - 0.119]	0.793	
		AD	-0.073 [-0.142 - -0.003]	**0.041**		-0.001 [-0.081 - 0.079]	0.98		-0.114 [-0.197 - -0.03]	**0.007**	*
	PASE	All	-0.066 [-0.11 - -0.021]	**0.004**	*	-0.008 [-0.062 - 0.046]	0.766		-0.048 [-0.098 - 0.002]	**0.06**	
		SCD	-0.025 [-0.068 - 0.018]	0.26		-0.012 [-0.073 - 0.049]	0.696		-0.054 [-0.134 - 0.026]	0.188	
		MCI	0.035 [-0.052 - 0.123]	0.425		-0.049 [-0.19 - 0.093]	0.498		-0.078 [-0.171 - 0.015]	0.102	
		AD	-0.072 [-0.147 - 0.002]	0.056		-0.017 [-0.095 - 0.061]	0.669		-0.042 [-0.126 - 0.042]	0.33	

Coefficients from linear mixed-effect models, adjusted for age, sex, **education (tertiles) and APOE- ε4 carriership.**
Analyses presented in this table were conducted exclusively within the amyloid-positive Alzheimer's Disease (AD) continuum sample.
Stars represent FDR adjusted p-values: * = p_{FDR} < 0.05, ** = p_{FDR} < 0.01, *** = p_{FDR} < 0.001

Supplementary Table 11a. Three-way interaction effect of cognitive/physical activity with temporoparietal cortical thickness on longitudinal rate of decline

			MMSE			Memory			Executive function			
			Estimate [CI]	p	pFDR	Estimate [CI]	p	pFDR	Estimate [CI]	p	pFDR	
β Time × TP thickness × Questionnaire score	Lifetime CAQ	All	-0.01 [-0.042 - 0.021]	0.519		0.005 [-0.03 - 0.04]	0.77		0.007 [-0.022 - 0.035]	0.641		
		SCD	-0.003 [-0.062 - 0.057]	0.924		0.003 [-0.075 - 0.081]	0.934		-0.007 [-0.127 - 0.113]	0.907		
		MCI	-0.015 [-0.078 - 0.048]	0.635		0.043 [-0.017 - 0.104]	0.159		0.057 [-0.008 - 0.122]	0.084		
		AD	-0.014 [-0.07 - 0.042]	0.62		-0.003 [-0.06 - 0.053]	0.908		0.011 [-0.072 - 0.094]	0.8		
	Past CAQ	All	-0.012 [-0.043 - 0.019]	0.438		0.001 [-0.033 - 0.035]	0.951		0 [-0.027 - 0.028]	0.982		
		SCD	0.001 [-0.053 - 0.056]	0.963		0.005 [-0.068 - 0.078]	0.896		-0.007 [-0.12 - 0.106]	0.908		
		MCI	-0.009 [-0.069 - 0.051]	0.768		0.047 [-0.01 - 0.105]	0.108		0.058 [-0.004 - 0.121]	0.069		
		AD	-0.025 [-0.08 - 0.03]	0.365		-0.016 [-0.073 - 0.041]	0.584		-0.024 [-0.106 - 0.057]	0.558		
	Current CAQ	All	0.006 [-0.026 - 0.038]	0.706		0.016 [-0.02 - 0.051]	0.383		0.026 [-0.005 - 0.057]	0.102		
		SCD	-0.026 [-0.102 - 0.05]	0.505		-0.009 [-0.104 - 0.086]	0.851		0.004 [-0.131 - 0.138]	0.956		
		MCI	-0.023 [-0.09 - 0.044]	0.504		0 [-0.067 - 0.068]	0.998		0.017 [-0.051 - 0.086]	0.623		
		AD	0.027 [-0.025 - 0.079]	0.311		0.031 [-0.021 - 0.083]	0.242		0.102 [0.024 - 0.179]	**0.01**		*
	PASE	All	-0.003 [-0.033 - 0.026]	0.82		0.01 [-0.023 - 0.043]	0.555		-0.003 [-0.028 - 0.022]	0.823		
		SCD	-0.002 [-0.054 - 0.051]	0.954		0.036 [-0.033 - 0.106]	0.306		0.002 [-0.107 - 0.111]	0.973		
		MCI	-0.053 [-0.109 - 0.003]	0.066		-0.035 [-0.09 - 0.019]	0.207		-0.014 [-0.073 - 0.044]	0.634		
		AD	0.026 [-0.028 - 0.08]	0.341		0.007 [-0.045 - 0.06]	0.789		-0.032 [-0.096 - 0.033]	0.334		

Coefficients from linear mixed-effect models, adjusted for age and sex.

Analyses presented in this table were conducted exclusively within the amyloid-positive Alzheimer's Disease (AD) continuum sample.

Stars represent FDR adjusted p-values: * = $p_{FDR} < 0.05$, ** = $p_{FDR} < 0.01$, *** = $p_{FDR} < 0.001$

Supplementary Table 11b. Three-way interaction effect of cognitive/physical activity with temporoparietal cortical thickness on longitudinal rate of decline, adjusted for education, operationalized as tertiles

			MMSE			Memory			Executive function		
			Estimate [CI]	p	pFDR	Estimate [CI]	p	pFDR	Estimate [CI]	p	pFDR
β Time × TP thickness × Questionnaire score	Lifetime CAQ	All	-0.009 [-0.041 - 0.023]	0.571		0.006 [-0.029 - 0.041]	0.73		0.009 [-0.019 - 0.038]	0.525	
		SCD	0.005 [-0.053 - 0.063]	0.87		0.007 [-0.072 - 0.086]	0.856		0.005 [-0.114 - 0.124]	0.929	
		MCI	-0.014 [-0.076 - 0.049]	0.669		0.044 [-0.017 - 0.105]	0.16		0.059 [-0.007 - 0.125]	0.079	
		AD	-0.014 [-0.07 - 0.042]	0.619		-0.006 [-0.063 - 0.05]	0.826		0.021 [-0.061 - 0.104]	0.612	
	Past CAQ	All	-0.011 [-0.043 - 0.02]	0.469		0.002 [-0.032 - 0.037]	0.897		0.002 [-0.025 - 0.03]	0.875	
		SCD	0.008 [-0.045 - 0.062]	0.767		0.009 [-0.065 - 0.083]	0.814		0.005 [-0.107 - 0.117]	0.926	
		MCI	-0.006 [-0.066 - 0.053]	0.834		0.048 [-0.01 - 0.107]	0.107		0.061 [-0.003 - 0.124]	0.061	
		AD	-0.027 [-0.082 - 0.028]	0.343		-0.015 [-0.072 - 0.042]	0.615		-0.01 [-0.092 - 0.071]	0.8	
	Current CAQ	All	0.008 [-0.024 - 0.04]	0.63		0.016 [-0.02 - 0.051]	0.393		0.029 [-0.003 - 0.06]	0.072	
		SCD	-0.018 [-0.092 - 0.056]	0.631		-0.006 [-0.102 - 0.09]	0.898		0.011 [-0.122 - 0.145]	0.866	
		MCI	-0.03 [-0.097 - 0.037]	0.38		-0.002 [-0.07 - 0.067]	0.965		0.015 [-0.053 - 0.084]	0.664	
		AD	0.031 [-0.022 - 0.083]	0.253		0.021 [-0.032 - 0.073]	0.439		0.102 [0.023 - 0.181]	**0.012**	*
	PASE	All	-0.002 [-0.032 - 0.027]	0.871		0.007 [-0.026 - 0.04]	0.674		-0.003 [-0.028 - 0.022]	0.803	
		SCD	-0.005 [-0.057 - 0.046]	0.835		0.032 [-0.038 - 0.103]	0.369		-0.009 [-0.117 - 0.099]	0.871	
		MCI	-0.057 [-0.115 - 0]	**0.049**		-0.033 [-0.091 - 0.025]	0.265		-0.021 [-0.082 - 0.041]	0.512	
		AD	0.026 [-0.028 - 0.079]	0.351		0.016 [-0.038 - 0.069]	0.571		-0.034 [-0.099 - 0.03]	0.296	

Coefficients from linear mixed-effect models, adjusted for age and sex and **education (tertiles).**
Analyses presented in this table were conducted exclusively within the amyloid-positive Alzheimer's Disease (AD) continuum sample.
Stars represent FDR adjusted p-values: * = $p_{FDR} < 0.05$, ** = $p_{FDR} < 0.01$, *** = $p_{FDR} < 0.001$

Supplementary Table 11c. Three-way interaction effect of cognitive/physical activity with temporoparietal cortical thickness on longitudinal rate of decline, adjusted for education, operationalized as (Verhage-transformed) years of education.

			MMSE			Memory			Executive function		
			Estimate [CI]	p	pFDR	Estimate [CI]	p	pFDR	Estimate [CI]	p	pFDR
β Time × TP thickness × Questionnaire score	Lifetime CAQ	All	-0.009 [-0.041 - 0.023]	0.584		0.005 [-0.03 - 0.04]	0.771		0.008 [-0.021 - 0.036]	0.599	
		SCD	-0.005 [-0.066 - 0.057]	0.879		-0.001 [-0.081 - 0.08]	0.984		-0.008 [-0.132 - 0.116]	0.902	
		MCI	-0.01 [-0.073 - 0.052]	0.744		0.045 [-0.016 - 0.106]	0.148		0.058 [-0.008 - 0.124]	0.087	
		AD	-0.013 [-0.069 - 0.043]	0.64		-0.005 [-0.061 - 0.051]	0.862		0.021 [-0.062 - 0.103]	0.621	
	Past CAQ	All	-0.011 [-0.042 - 0.02]	0.485		0.001 [-0.033 - 0.035]	0.95		0.001 [-0.027 - 0.028]	0.948	
		SCD	0 [-0.056 - 0.057]	0.988		0.002 [-0.073 - 0.077]	0.959		-0.006 [-0.123 - 0.111]	0.919	
		MCI	-0.004 [-0.064 - 0.057]	0.905		0.049 [-0.009 - 0.107]	0.098		0.059 [-0.005 - 0.122]	0.071	
		AD	-0.025 [-0.081 - 0.03]	0.364		-0.013 [-0.07 - 0.044]	0.656		-0.011 [-0.092 - 0.07]	0.792	
	Current CAQ	All	0.007 [-0.025 - 0.039]	0.68		0.017 [-0.019 - 0.052]	0.36		0.028 [-0.004 - 0.059]	0.082	
		SCD	-0.029 [-0.106 - 0.048]	0.453		-0.014 [-0.111 - 0.082]	0.773		0.001 [-0.136 - 0.139]	0.984	
		MCI	-0.025 [-0.092 - 0.042]	0.472		0 [-0.068 - 0.067]	0.992		0.017 [-0.052 - 0.085]	0.634	
		AD	0.028 [-0.024 - 0.08]	0.29		0.021 [-0.032 - 0.073]	0.439		0.096 [0.019 - 0.174]	**0.015**	*
	PASE	All	-0.003 [-0.033 - 0.027]	0.841		0.009 [-0.024 - 0.042]	0.603		-0.003 [-0.028 - 0.022]	0.821	
		SCD	-0.002 [-0.055 - 0.051]	0.95		0.034 [-0.036 - 0.105]	0.337		-0.003 [-0.114 - 0.107]	0.951	
		MCI	-0.051 [-0.107 - 0.005]	0.072		-0.034 [-0.09 - 0.021]	0.22		-0.013 [-0.072 - 0.045]	0.655	
		AD	0.026 [-0.027 - 0.08]	0.338		0.01 [-0.042 - 0.063]	0.7		-0.03 [-0.094 - 0.034]	0.36	

Coefficients from linear mixed-effect models, adjusted for age and sex and **education (years).**

Analyses presented in this table were conducted exclusively within the amyloid-positive Alzheimer's Disease (AD) continuum sample.

Stars represent FDR adjusted p-values: * = $p_{FDR} < 0.05$, ** = $p_{FDR} < 0.01$, *** = $p_{FDR} < 0.001$

Supplementary Table 11d. Three-way interaction effect of cognitive/physical activity with temporoparietal cortical thickness on longitudinal rate of decline, adjusted for education, operationalized as tertiles, and for APOE-ε4 carriership status

			MMSE			Memory			Executive function		
			Estimate [CI]	p	pFDR	Estimate [CI]	p	pFDR	Estimate [CI]	p	pFDR
β Time × TP thickness × Questionnaire score	Lifetime CAQ	All	-0.009 [-0.042 - 0.023]	0.562		0.007 [-0.028 - 0.042]	0.703		0.01 [-0.019 - 0.038]	0.508	
		SCD	0.008 [-0.051 - 0.067]	0.795		0.024 [-0.055 - 0.103]	0.557		-0.002 [-0.124 - 0.119]	0.972	
		MCI	-0.014 [-0.076 - 0.049]	0.668		0.042 [-0.019 - 0.104]	0.175		0.057 [-0.008 - 0.122]	0.086	
		AD	-0.015 [-0.071 - 0.041]	0.601		-0.006 [-0.062 - 0.05]	0.832		0.021 [-0.061 - 0.103]	0.619	
	Past CAQ	All	-0.012 [-0.043 - 0.019]	0.463		0.003 [-0.032 - 0.037]	0.879		0.002 [-0.025 - 0.03]	0.861	
		SCD	0.01 [-0.044 - 0.064]	0.718		0.022 [-0.052 - 0.096]	0.565		-0.001 [-0.115 - 0.113]	0.987	
		MCI	-0.007 [-0.066 - 0.053]	0.83		0.047 [-0.012 - 0.105]	0.12		0.059 [-0.004 - 0.122]	0.068	
		AD	-0.027 [-0.082 - 0.028]	0.336		-0.015 [-0.072 - 0.042]	0.603		-0.01 [-0.091 - 0.071]	0.805	
	Current CAQ	All	0.007 [-0.025 - 0.039]	0.653		0.016 [-0.019 - 0.052]	0.364		0.029 [-0.002 - 0.061]	0.065	
		SCD	-0.012 [-0.089 - 0.064]	0.754		0.02 [-0.077 - 0.118]	0.685		0.001 [-0.137 - 0.138]	0.994	
		MCI	-0.03 [-0.097 - 0.038]	0.39		0 [-0.069 - 0.068]	0.99		0.016 [-0.052 - 0.084]	0.648	
		AD	0.029 [-0.024 - 0.082]	0.278		0.023 [-0.03 - 0.076]	0.391		0.1 [0.021 - 0.18]	**0.013**	*
	PASE	All	-0.003 [-0.033 - 0.027]	0.839		0.007 [-0.026 - 0.04]	0.667		-0.004 [-0.029 - 0.021]	0.75	
		SCD	-0.008 [-0.06 - 0.044]	0.769		0.021 [-0.05 - 0.093]	0.56		-0.005 [-0.115 - 0.106]	0.932	
		MCI	-0.058 [-0.115 - 0]	0.05		-0.033 [-0.091 - 0.025]	0.258		-0.024 [-0.085 - 0.037]	0.437	
		AD	0.024 [-0.03 - 0.078]	0.388		0.015 [-0.038 - 0.069]	0.576		-0.034 [-0.099 - 0.031]	0.301	

Coefficients from linear mixed-effect models, adjusted for age, sex, **education (tertiles) and APOE- ε4 carriership.**
Analyses presented in this table were conducted exclusively within the amyloid-positive Alzheimer's Disease (AD) continuum sample.
Stars represent FDR adjusted p-values: * = $p_{FDR} < 0.05$, ** = $p_{FDR} < 0.01$, *** = $p_{FDR} < 0.001$

Supplementary Table 12a. Risk of progression and of mortality, adjusted for education operationalized as tertiles

		Mortality			Clinical progression to MCI/Dementia		
		HR [CI]	p	pFDR	HR [CI]	p	pFDR
Lifetime CAQ	All	0.92 [0.81 - 1.05]	0.24		1.1 [0.87 - 1.39]	0.41	
	SCD	1.16 [0.53 - 2.53]	0.66		1.06 [0.69 - 1.63]	0.79	
	MCI	0.56 [0.35 - 0.88]	**0.01**	*	1.06 [0.8 - 1.39]	0.69	
	AD	0.98 [0.85 - 1.14]	0.82		-	-	-
Past CAQ	All	0.96 [0.84 - 1.1]	0.57		1.15 [0.92 - 1.45]	0.22	
	SCD	1.18 [0.55 - 2.54]	0.62		1.08 [0.71 - 1.64]	0.71	
	MCI	0.57 [0.37 - 0.87]	**0.01**	*	1.1 [0.84 - 1.45]	0.48	
	AD	1.02 [0.89 - 1.18]	0.76		-	-	-
Current CAQ	All	0.84 [0.74 - 0.96]	**0.01**	**	0.88 [0.69 - 1.12]	0.3	
	SCD	1.04 [0.38 - 2.87]	0.93		0.93 [0.56 - 1.56]	0.78	
	MCI	0.85 [0.55 - 1.32]	0.46		0.87 [0.65 - 1.16]	0.33	
	AD	0.88 [0.76 - 1.01]	0.07		-	-	-
PASE	All	0.89 [0.79 - 1]	**0.04**	*	0.94 [0.77 - 1.14]	0.5	
	SCD	0.87 [0.31 - 2.49]	0.76		0.91 [0.59 - 1.41]	0.67	
	MCI	1 [0.73 - 1.37]	0.99		0.92 [0.73 - 1.16]	0.47	
	AD	0.87 [0.76 - 0.99]	**0.03**	*	-	-	-

Hazard ratios (HR) and 95% Confidence Intervals (CI) from Cox regression models. adjusted for age, sex and **education** (**tertiles**)
Analyses presented in this table were conducted exclusively within the amyloid-positive Alzheimer's Disease (AD) continuum sample.
Stars represent FDR adjusted p-values: * = $p_{FDR} < 0.05$, ** = $p_{FDR} < 0.01$, *** = $p_{FDR} < 0.001$

Supplementary Table 12b. Risk of progression and of mortality, adjusted for education, operationalized as (Verhage-transformed) years of education.

		Mortality			Clinical progression to MCI/Dementia		
		HR [CI]	p	pFDR	HR [CI]	p	pFDR
Lifetime CAQ	All	0.95 [0.83 - 1.08]	0.43		1.08 [0.87 - 1.36]	0.47	
	SCD	1.24 [0.57 - 2.66]	0.52		1.01 [0.66 - 1.54]	0.96	
	MCI	0.57 [0.36 - 0.9]	**0.02**	*	1.05 [0.8 - 1.37]	0.74	
	AD	1 [0.86 - 1.15]	0.95		-	-	-
Past CAQ	All	0.99 [0.87 - 1.12]	0.82		1.13 [0.91 - 1.4]	0.27	
	SCD	1.24 [0.58 - 2.64]	0.51		1.03 [0.68 - 1.56]	0.88	
	MCI	0.58 [0.38 - 0.89]	**0.01**	*	1.09 [0.83 - 1.42]	0.53	
	AD	1.03 [0.9 - 1.19]	0.64		-	-	-
Current CAQ	All	0.86 [0.76 - 0.98]	**0.02**	*	0.88 [0.69 - 1.12]	0.29	
	SCD	1.21 [0.43 - 3.37]	0.66		0.91 [0.55 - 1.5]	0.69	
	MCI	0.87 [0.56 - 1.35]	0.52		0.86 [0.65 - 1.15]	0.32	
	AD	0.89 [0.77 - 1.02]	0.09		-	-	-
PASE	All	0.88 [0.78 - 0.99]	**0.03**	*	0.92 [0.76 - 1.12]	0.41	
	SCD	0.95 [0.34 - 2.67]	0.9		0.9 [0.58 - 1.39]	0.61	
	MCI	0.98 [0.71 - 1.34]	0.88		0.92 [0.73 - 1.16]	0.47	
	AD	0.87 [0.76 - 0.99]	**0.03**	*	-	-	-

Hazard ratios (HR) and 95% Confidence Intervals (CI) from Cox regression models. adjusted for age, sex and **education (years)**
Analyses presented in this table were conducted exclusively within the amyloid-positive Alzheimer's Disease (AD) continuum sample.
Stars represent FDR adjusted p-values: * = $p_{FDR} < 0.05$, ** = $p_{FDR} < 0.01$, *** = $p_{FDR} < 0.001$

Supplementary Table 12c. Risk of progression and of mortality, adjusted for education, operationalized as tertiles, and for APOE-ε4 carriership status

		Mortality			Clinical progression to MCI/Dementia		
		HR [CI]	p	pFDR	HR [CI]	p	pFDR
Lifetime CAQ	All	0.92 [0.81 - 1.05]	0.23		1.12 [0.89 - 1.41]	0.33	
	SCD	1.16 [0.5 - 2.68]	0.66		1.12 [0.72 - 1.75]	0.6	
	MCI	0.53 [0.33 - 0.85]	**0.01**	*	1.06 [0.8 - 1.39]	0.69	
	AD	0.98 [0.85 - 1.14]	0.83		-	-	-
Past CAQ	All	0.96 [0.84 - 1.1]	0.55		1.17 [0.93 - 1.47]	0.17	
	SCD	1.18 [0.52 - 2.69]	0.62		1.13 [0.74 - 1.73]	0.55	
	MCI	0.53 [0.34 - 0.83]	**0.01**	**	1.1 [0.84 - 1.45]	0.48	
	AD	1.02 [0.89 - 1.18]	0.75		-	-	-
Current CAQ	All	0.85 [0.75 - 0.96]	**0.01**	**	0.89 [0.7 - 1.13]	0.33	
	SCD	1.02 [0.34 - 3.07]	0.97		1.01 [0.59 - 1.74]	0.96	
	MCI	0.86 [0.55 - 1.33]	0.48		0.87 [0.65 - 1.16]	0.34	
	AD	0.88 [0.76 - 1.01]	0.07		-	-	-
PASE	All	0.89 [0.79 - 1]	**0.04**	*	0.93 [0.76 - 1.13]	0.46	
	SCD	0.85 [0.27 - 2.69]	0.72		0.87 [0.56 - 1.37]	0.54	
	MCI	1 [0.73 - 1.37]	0.99		0.92 [0.73 - 1.16]	0.48	
	AD	0.87 [0.76 - 0.99]	**0.03**	*	-	-	-

Hazard ratios (HR) and 95% Confidence Intervals (CI) from Cox regression models, adjusted for age, sex, **education (tertiles) and APOE- ε4 carriership.**

Analyses presented in this table were conducted exclusively within the amyloid-positive Alzheimer's Disease (AD) continuum sample.

Stars represent FDR adjusted p-values: * = $p_{FDR} < 0.05$, ** = $p_{FDR} < 0.01$, *** = $p_{FDR} < 0.001$
